# Associations between use of macrolide antibiotics during pregnancy and adverse child outcomes: A systematic review and meta-analysis

**DOI:** 10.1371/journal.pone.0212212

**Published:** 2019-02-19

**Authors:** Heng Fan, Leah Li, Linda Wijlaars, Ruth E. Gilbert

**Affiliations:** 1 Population, Policy and Practice Programme, Great Ormond Street Institute of Child Health, University College London, London, United Kingdom; 2 Administrative Data Research Centre for England, University College London, London, United Kingdom; University of Milano-Bicocca, ITALY

## Abstract

**Background:**

Evidence on adverse effects of maternal macrolide use during pregnancy is inconsistent. We conducted a systematic review and meta-analysis to investigate the association between macrolide use during pregnancy and adverse fetal and child outcomes.

**Methods and findings:**

We included observational studies and randomised controlled trials (RCTs) that recorded macrolide use during pregnancy and child outcomes. We prioritized comparisons of macrolides with alternative antibiotics (mainly penicillins or cephalosporins) for comparability of indication and effect. Random effects meta-analysis was used to derive pooled odds ratios (OR) for each outcome. Subgroup analyses were performed according to specific types (generic forms) of macrolide.

Of 11,186 citations identified, 19 (10 observational, 9 RCTs) studies were included (21 articles including 228,556 participants). Macrolide prescribing during pregnancy was associated with an increased risk of miscarriage (pooled OR_obs_ 1·82, 95% CI 1·57–2·11, three studies, I^2^ = 0%), cerebral palsy and/or epilepsy (OR_obs_ 1·78, 1·18–2·69; one study), epilepsy alone (OR_obs_ 2·02, 1·30–3·14, one study; OR_RCT_ 1.03, 0.79–1.35, two studies), and gastrointestinal malformations (OR_obs_ 1·56, 1·05–2·32, two studies) compared with alternative antibiotics. We found no evidence of an adverse effect on 12 other malformations, stillbirth, or neonatal death. Results were robust to excluding studies with high risk of bias.

**Conclusions:**

Consistent evidence of an increased risk of miscarriage in observational studies and uncertain risks of cerebral palsy and epilepsy warrant cautious use of macrolide in pregnancy with warnings in drug safety leaflets and use of alternative antibiotics where appropriate. As macrolides are the third most commonly used class of antibiotics, it is important to confirm these results with high quality studies.

## Introduction

Macrolide antibiotics are one of the most commonly used class of antibiotics worldwide. [1, 2]. Major members of the class include erythromycin, azithromycin and clarithromycin. Over the last 20 years, concerns have been raised about rare but serious adverse outcomes associated with macrolide use during pregnancy [3–6]. The strongest evidence comes from a large randomised controlled trial (RCT, ORACLE Child Study II) of women with spontaneous preterm labour (SPL), which reported an increased risk of cerebral palsy in children whose mothers received erythromycin (3·3%) compared with no erythromycin (1·7%, Odds Ratio (OR) 1·93, 95% Confidence Interval (CI): 1·21–3·09) [6]. Increased risks of miscarriage, major malformations, and cardiovascular malformation have also been reported in some observational studies [3, 7–10], but not in others [11–13]. Currently there is no overview of the effects of macrolide treatment during pregnancy in fetuses or children and no consensus about whether macrolides are considered safe in pregnancy or not. In April 2015, the UK Medicines and Healthcare products Regulatory Agency (MHRA) reviewed evidence indicating potential harmful effects of macrolides on cerebral palsy or epilepsy reported by Meeraus et al [5], and decided there was insufficient evidence to warn against macrolides use in pregnancy [14]. However, in Sweden, 2005, national policy advised against the use of erythromycin during early pregnancy [9]. Warnings have been also issued against use of azithromycin and clarithromycin in adults with high risk of cardiovascular complications in the United States (US) [15, 16], based on evidence of an unexpected increase in the risk of death and cardiovascular events including arrhythmias and cardiac mortality (Azithromycin: 2 systematic reviews including 20 RCTs [17, 18]; clarithromycin: 1 RCT [19]).

Interpretation of existing research is challenging. In observational studies, the harm observed after macrolides antibiotics exposure could be a consequence of infection–rather than the use of macrolides (i.e. indication bias). On the other hand, RCTs are usually not design to look at adverse event. In RCTs that compare macrolides with placebo, a “real” harmful effect of macrolides could be masked due to its benefits of treating infection and thus only a null or protective effect can be observed.

We conducted a systematic review and meta-analysis to determine the effects of macrolide treatment during pregnancy on fetal and child outcomes. We prioritized comparisons between macrolides and alternative antibiotics (mainly penicillins or cephalosporins) for assumed comparability of indication and treatment effects. RCTs where one of the arms included placebo or drug combinations (e.g. erythromycin plus co-amoxiclav) are susceptible to incomparable treatment effects and were therefore regarded as secondary comparisons. Based on previous evidence from experimental and epidemiological studies, we hypothesized that short-term fetal hypoxia induced by fetal arrhythmia could possibly be the underlying mechanism of observed adverse effects of macrolides (S1 Text). We therefore included outcomes which could potentially result from short-term fetal hypoxia (i.e. fetal and neonatal death, congenital malformations, and conditions resulting from central nervous system damage). We presented pooled effects of macrolides use in pregnancy on each outcome and explored heterogeneity of the effect according to specific types of macrolides.

## Methods

A complete PRISMA harm checklist for this review is available in S1 Table. A protocol for this review has not been previously published.

### Eligibility criteria

#### Study types

We sought comparative studies which examined macrolide treatment during pregnancy and adverse fetal and/or childhood outcomes that have been reported to be associated with fetal hypoxia. We included randomised controlled trials and observational (cohort or case-control) studies and set different eligibility criteria for the comparator group according to study type in order to address the risk of bias as explained below.

#### Participants, interventions and comparisons

The exposed populations were the fetuses or children whose mothers were prescribed macrolide antibiotics during pregnancy. We included studies that included all pregnancies or those that reported outcomes only in live-births.

The primary analysis included studies comparing macrolide antibiotics with alternative antibiotics e.g. penicillin or cephalosporin. Macrolides are recommended as the alternative for women with suspected allergy to penicillin or cephalosporin, thereby minimising the risk of indication bias due to infection [20, 21]. Penicillin and cephalosporin are also comparable with macrolides in treatment effect with long-established safety records during pregnancy [21]. This head to head comparison thus also allows the separation of possible harm of macrolides from their benefits of treatment on infection.

The secondary analysis includes RCTs with the following two comparisons: macrolide versus placebo and macrolides plus alternative antibiotics versus the same alternatives. Though RCTs avoid indication bias by design, true adverse effects of macrolides may be masked by the benefits of macrolides in reducing infection, thereby underestimating adverse effects.

#### Outcomes

We reviewed cohort studies of pregnant women and their children and experimental studies in animals to identify outcomes that could potentially result from short term fetal hypoxia, including fetal and neonatal death, congenital malformations, and conditions resulting from central nervous system damage (i.e. epilepsy, cerebral palsy, ADHD and autism) [21–23]. We excluded outcomes commonly related to chronic hypoxia (e.g. low birthweight, intrauterine growth restriction) or outcomes that might result from postnatal events (e.g. abnormal cerebral ultrasonography).

### Search strategy

Systematic literature searches were conducted in PubMed, Embase, Cochrane Library, Conference Proceeding Citation Index-Science and ClinicalTrials.gov from their respective inception until Februry 15, 2018 (S2 Table). We included conference abstracts if sufficient data were provided. Further relevant studies were retrieved by hand searching the reference lists of eligible papers and by using ‘Similar articles’ function within databases. No language restrictions were applied.

### Study selection

After removal of duplicates, the titles and abstracts of all identified records (full-text articles and abstracts) were evaluated by reviewer HF, using a “decision tree” (S3 Table). Ten percent of the records were also reviewed by another reviewer LW, and an inter-rater agreement was calculated. A total of 967 studies were double reviewed with an inter-rater agreement of 81%. All studies potentially meeting the inclusion criteria were reviewed in full by reviewer HF, and 10% of the potentially eligible studies were reviewed by a third reviewer (LL). We resolved any discrepancies through discussion. Reviewers were not blinded to authors, journals or institutions.

### Data extraction

We developed and piloted a data extraction sheet on 15 relevant studies. Reviewer HF extracted relevant data from included studies. We contacted authors of potentially eligible manuscripts by email for relevant data if this could not be extracted from the publication. Where the same cohort was reported more than once, we extracted data from the study with the largest sample size.

For studies reporting multiple comparisons of each specific type of macrolides (vs same comparator), we divided the comparator group equally for each comparison. For studies presenting multiple estimates of exposure on both whole pregnancy and the first trimester, the estimate of exposure on the first trimester was included in the meta-analysis to avoid a potential dilution effect.

### Risk of bias assessment

We used risk of bias assessment tools for RCTs (Cochrane Collaboration’s tool for assessing risk of bias in randomised trials) and observational studies (Risk of Bias In Non-Randomised Studies–of Interventions (ROBIN-I)). The tool for RCTs was modified by including sections on masked adverse effect (susceptible when one of the arms was placebo or drug combinations, as mentioned in the eligibility criteria). We considered observational comparisons of macrolides versus alternative antibiotics to be at low or moderate risk of indication bias, provided there is no evidence of macrolides indication apart from that of alternative antibiotics (S4 and S5 Tables) [24, 25].

### Data synthesis and analysis

We estimated the pooled ORs for each adverse outcome using a random-effects meta-analysis, considering the heterogeneity among studies which was measured by *I*^2^ statistics. RCTs and observational studies were analysed separately. In the primary analysis, we compared macrolides with alternative antibiotics in RCTs and observational studies. In the secondary analyses, we included RCTs which compared (1) macrolides with alternative antibiotics, (2) macrolides with placebo and (3) macrolides plus alternative antibiotics with the same alternatives. We summarized the results according to specific types of macrolide in subgroup analysis. Sensitivity analysis was performed according to risks of bias.

Studies with no observed events were excluded from the meta-analysis. For studies with no events in one of the two groups, we applied a correction proportional to the reciprocal of the size of the contrasting study arm [26]. For observational studies, we used the adjusted OR if reported, otherwise, we re-calculated the crude OR using the data reported. Funnel plot asymmetry was not assessed because of an insufficient number of studies. Analyses were performed using R 3·4·1 (R Foundation for Statistical Computing, Vienna, Austria).

### Role of funding source

The funders of this study had no role in study design, data collection, analysis and interpretation, or writing of the report. The corresponding author had full access to all the data in the study and had final responsibility for the decision to submit for publication.

## Results

**Fig 1:**
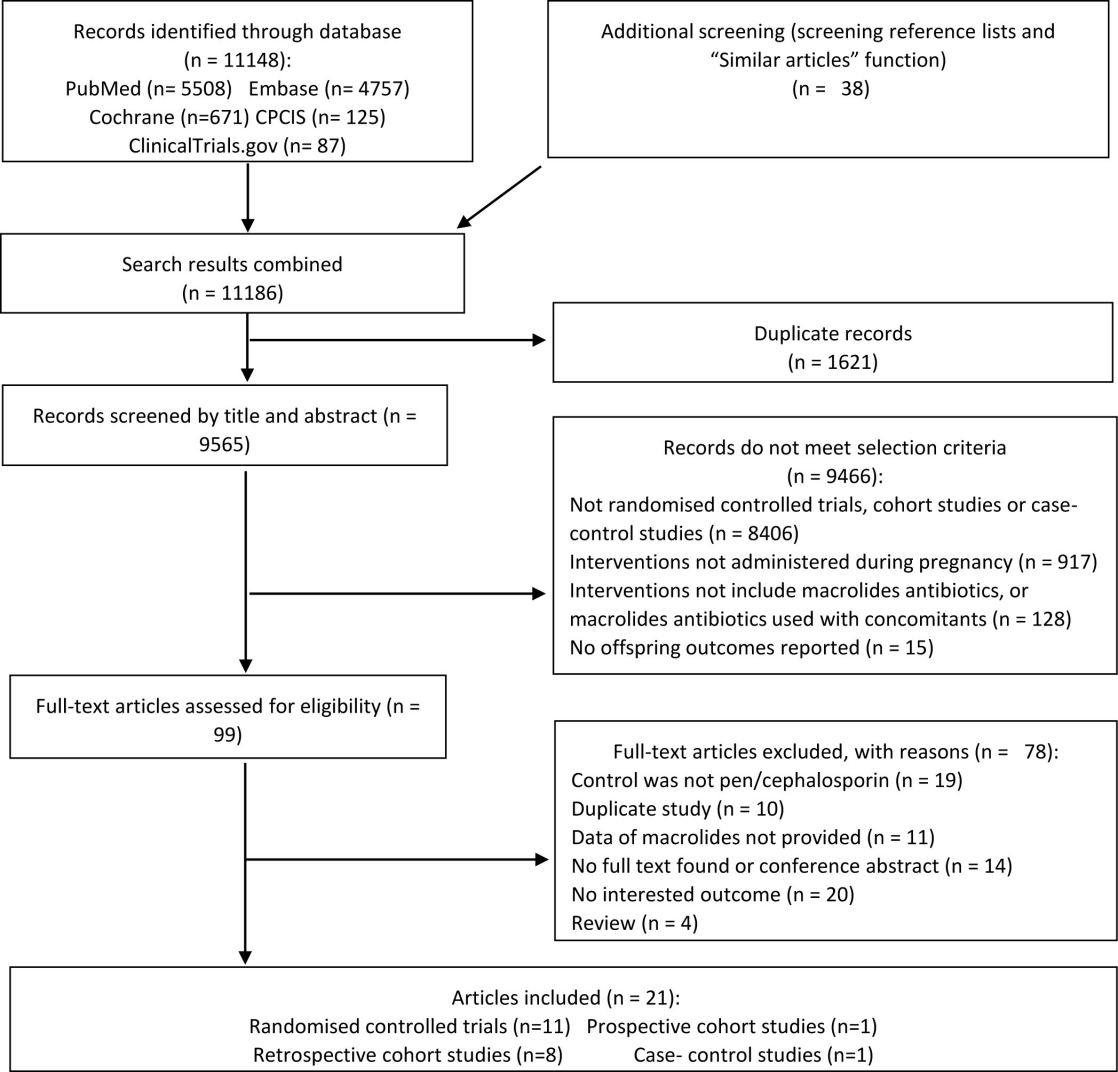
Study selection.

Of the 11,186 citations identified, we selected 99 abstracts for detailed assessment (Fig 1). 14 articles based on 12 studies (2 RCTs and 10 observational studies, 190,368 pregnancies or live births) met our inclusion criteria for primary analysis. Secondary analysis included 9 RCTs published in 11 articles of 15,405 pregnancies. Characteristics of the included studies are listed in S6 Table [3–8, 10, 13, 27–39].

**Fig 2:**
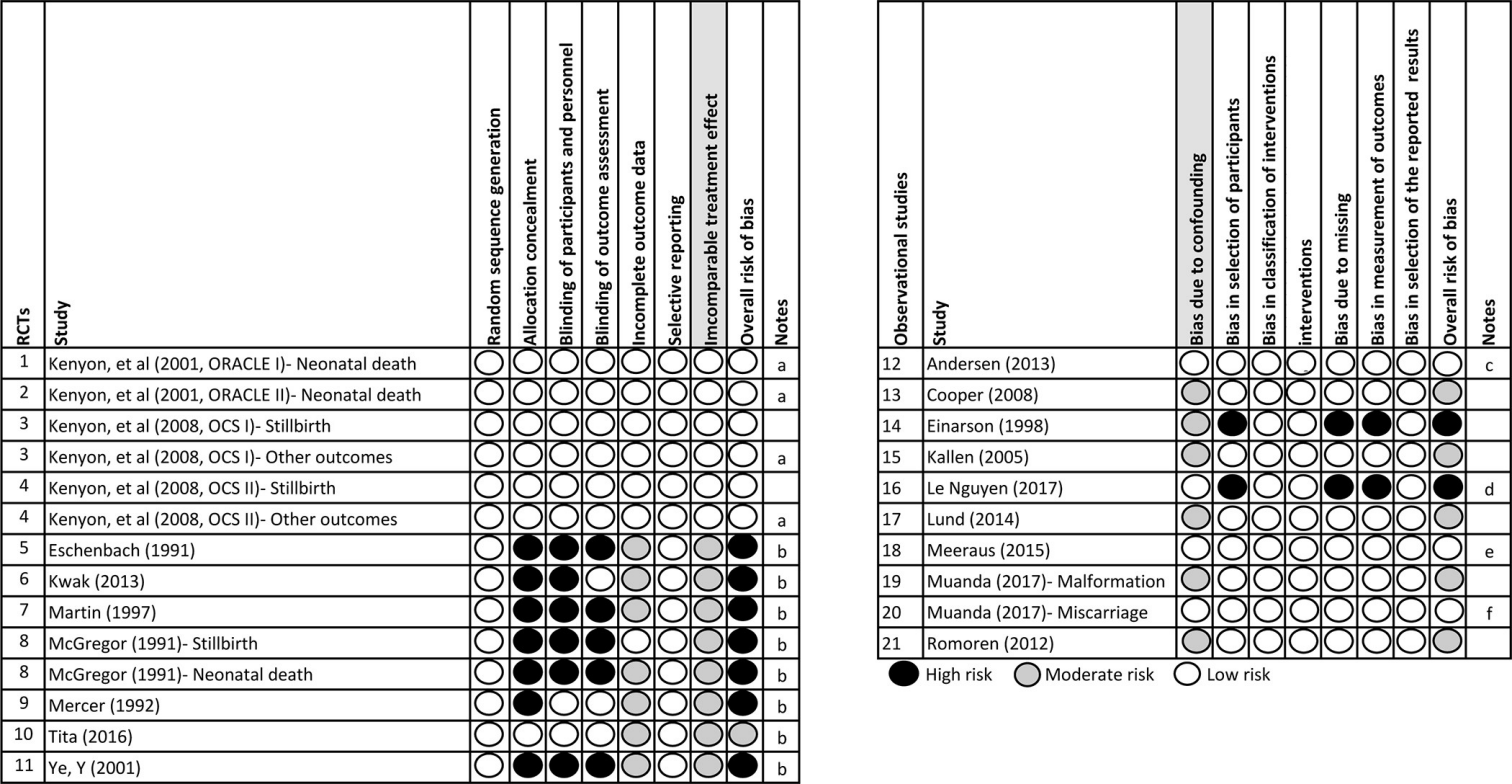
Assessment of bias. a: Evaluated as moderate risk in secondary analysis (low risk in primary analysis) due to incomparable treatment effect. b: Studies only eligible for secondary analysis. c: In the study of Andersen (2013), the OR was adjusted by maternal age, number of previous miscarriages, income and education. d: In the study of Le guyen, the OR was adjusted by maternal age, long-term illnesses, parity and multiple pregnancy. e: In the study of Meeraus (2015), the Hazard Ratio was adjusted by maternal age, Townsend quintile, year of delivery, smoking, alcohol problems, obesity, illicit drug use, treatment of chronic medical conditions and potentially neurologically-damaging infection during pregnancy. f: In the study of Muanda (2017), cases and controls were matched by gestational age and year of pregnancy; in the analysis of specific macrolides, the ORs were adjusted by 11 covariates, e.g. maternal age, education level, chronic comorbidities, maternal infections (urinary tract infection, respiratory tract infection, bacterial vaginosis and sexually transmitted infections) and prior exposure to antibiotics.

Overall, 12/14 (85·7%) articles in the primary analysis were judged to have low or medium overall risk of bias (Fig 2). Adjusted ORs were available for four of ten observational studies [5, 7, 8, 10].

**Fig 3:**
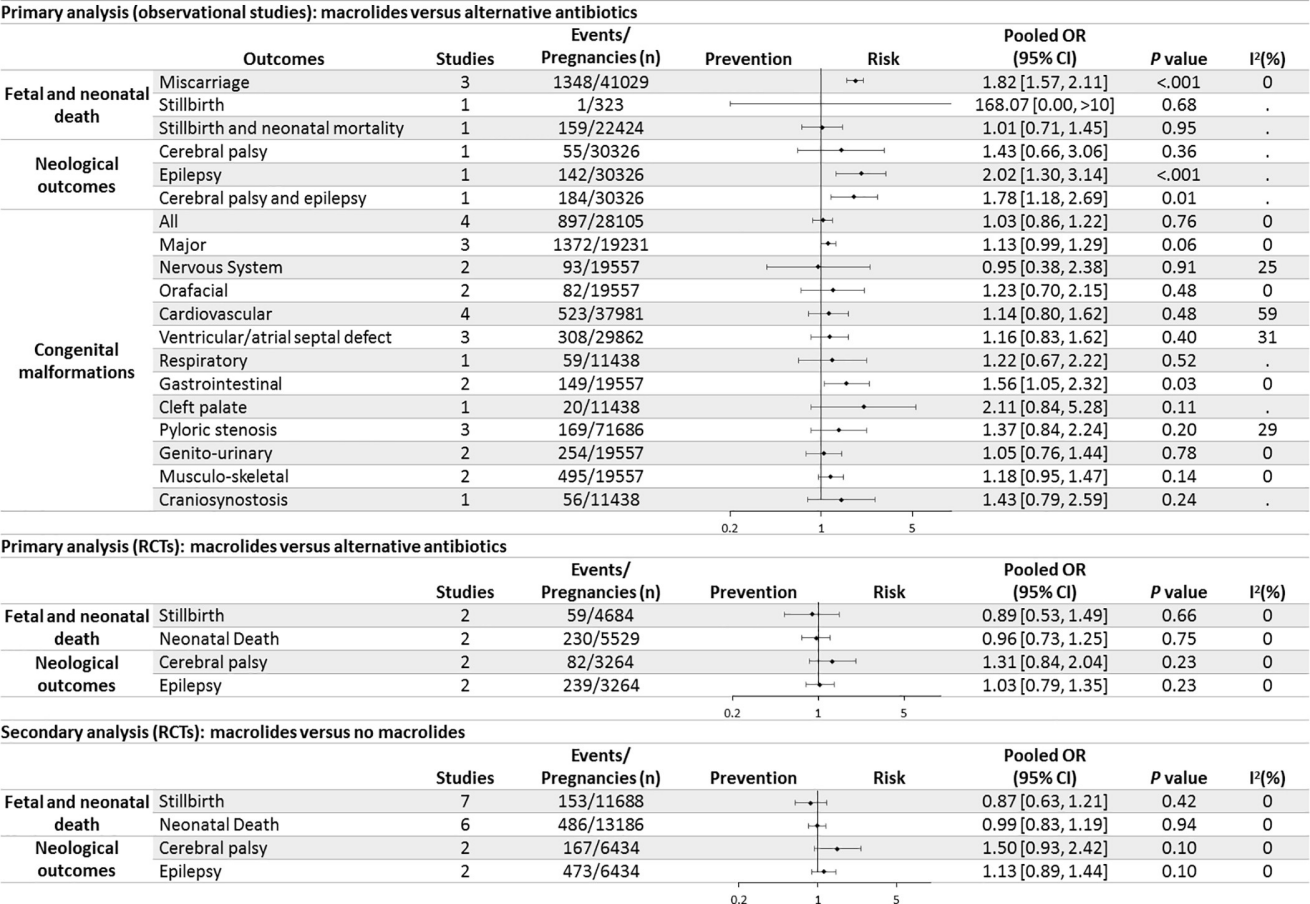
Primary and secondary analysis for the association between prenatal use of macrolides and adverse child outcomes.

All three observational studies in the primary analysis reporting the risk of miscarriage in the primary analysis showed an increased odd of miscarriage for mothers prescribed macrolide antibiotics compared with those prescribed alternative antibiotics, with a pooled OR of 1·82 (95% CI 1·57–2·11) (Fig 3 and S2 Fig) [3, 7, 8]. No RCTs included evaluated the risk of miscarriage. Given the lowest risk ratio estimate in one of the three studies as 1·51, the number needed to harm (NNH) for miscarriage ranges from 10 at 6 weeks’ gestation to 196 at 20 weeks, with the baseline risks of 20% and 1%, respectively [7, 40]. Compared with alternative antibiotics, odds of miscarriage increased significantly in subgroups prescribed azithromycin and clarithromycin, but not in the subgroup prescribed erythromycin (S3 Fig).

Two RCTs (4 articles) and 2 observational studies reported the association between macrolides use in pregnancy and stillbirth, neonatal death or “stillbirth and neonatal mortality” [3, 6, 13, 29–31]. The odds of these outcomes did not differ between macrolides and alternative antibiotics prescription. (Fig 3, S2 and S4 Figs).

No increase was noted in risks for cerebral palsy or epilepsy in 2 RCTs, one of women with SPL and the other of women with preterm, prelabour rupture of fetal membranes (pPROM) [6, 29]. However, in one large cohort study of Meeraus et al, the odds for epilepsy and a composite outcome “cerebral palsy and/or epilepsy” increased significantly in unselected mothers prescribed to macrolides compared with penicillins, with an NNH of 245 and 214, given the baseline risks of 0.4% and 0.6%, respectively (Fig 3, S2 and S4 Figs) [5, 41].

Seven observational studies evaluated the risk of malformations. No difference in risk was identified for “all malformations”, major malformations, cardiovascular malformations and pyloric stenosis. The results of other organ-specific malformations were dominated by one study of Muanda et al [36]. A significant but weak increase in the risk of gastrointestinal congenital malformations was observed in mothers prescribed macrolide antibiotics (with the NNH of 744, given a baseline incidence of 2·4 per 1000 live births) [42]. Evidence for specific types of macrolides was limited. Azithromycin was significantly associated with major, gastrointestinal and muscular-skeletal malformations. Erythromycin was the only macrolide significantly associated with orofacial malformations and cleft palate or cleft lip (Fig 3, S2 and S3 Figs).

Secondary analyses of RCTs with one arm as drug combinations or placebo showed similar results to those from the primary analysis [28, 32–35, 37, 38]. (S5 Fig). In the sensitivity analysis, restriction of analyses to comparisons with low or moderate risk of bias (applicable to miscarriage, “all malformation” and major malformation) did not alter the conclusions of primary analysis (S7 Fig).

## Discussion

We report a systematic review and meta-analysis of associations between maternal macrolide antibiotics exposure and adverse child outcomes. Macrolide antibiotics use during pregnancy (vs alternative antibiotics) was associated with an increased risk of miscarriage, but evidence for its association with cerebral palsy and epilepsy were inconsistent. We found only weak evidence for an association with gastrointestinal malformations and insufficient evidence with other malformations, stillbirth or neonatal death.

To our knowledge, this review is the first systematic assessment of the adverse fetal and child outcomes of macrolides use during pregnancy. The increased risk of miscarriage and inconsistent evidence for cerebral palsy and epilepsy suggests that macrolides may have the potential to cause adverse effects when used in pregnancy. Evidence from experimental and epidemiological studies in adults also indicates biological plausibility of embryotoxicity and cardiotoxity of exposure to azithromycin and clarithromycin (S1 Text). As the aetiology of these fetal adverse outcomes is multifactorial, the increase of absolute risk can be small for some conditions. Dependencies between comparisons may exist when we split the comparator groups and evaluated more than one outcomes from one study. Very few studies were available for each outcome, which resulted in imprecision in the heterogeneity estimates.

To address potential bias due to treatment indication, in the primary analysis we included only head-to-head comparisons, most of which (42 out of 47 comparisons, 89%) were between macrolides and penicillins. Macrolides are often used as replacement therapy for patients with penicillin allergy [20]. There are unique indications of macrolides (e.g. azithromycin for gonorrhoea, chlamydia and mycoplasma infection) which could be linked to increased risk of miscarriage [43]. However, the indications of clarithromycin do not include genitourinary tract infections according to guidelines in both Europe and North America [44–47]. Yet all three studies in this review reported consistent increased risks of miscarriage associated with clarithromycin [3, 7, 8]. Furthermore, it is estimated that over 20% penicillins prescribed at pregnancy are for genitourinary tract infection [1]. Therefore the increased risk of miscarriage observed in this review is unlikely to be over-estimated due to indications of macrolides. Among the three studies on miscarriage, two are large studies using Canadian and Danish registries. The Canadian study was a good-quality nested case-control study matched by gestational age, with adjustment for types of maternal infections (including sexually transmitted infections) and proxies for infection severity [8]. Indication was also analysed in the Danish study by comparing clarithromycin with erythromycin, phenoxymethylpenicillin, amoxicillin and proton pump inhibitors adjusted by maternal characteristics, with consistent evidence of increased risks of miscarriage in the clarithromycin group (S6 Table) [7].

Another source of bias in studies on outcomes that manifest after birth (e.g. cerebral palsy, epilepsy and malformations) is survivor bias. Assuming an increased risk of fetal death (i.e. miscarriage) in fetuses exposed to macrolides (vs penicillin), both fetuses exposed to macrolides and fetuses with adverse outcome were less likely to be selected in studies conditioning on live-birth status. Thereby this survivor bias would weaken the association between macrolides and adverse outcomes in live-born children. The observed risk ratios for gastrointestinal malformation, cerebral palsy and epilepsy in this review may therefore be underestimated due to survivor bias. The potential effects of survivor bias is illustrated by a post-hoc simulation in S2 Text.

Heterogeneity exists among studies due to study design (RCT or observational), population of pregnant women studied, specific types of macrolides, and gestational ages for administering macrolides. Our analyses of RCTs were dominated by studies of mothers at high risk of fetal infection, e.g. mothers with pPROM or urinary tract infections. Comparing with placebo, these mothers stand to benefit from the treatment effect of macrolides, masking evidence of its potential harm. However, in mothers with SPL, who bore a relatively low risk of fetal infection, masked harm from macrolides due to its treatment benefits is reduced. Reduced risk of fetal infection may explain the finding of significantly increased risk of cerebral palsy in mothers with SPL, but not in mothers with pPROM in secondary analysis (S5 Fig).

Evidence of heterogeneity in adverse outcomes according to specific types of macrolides is limited because of few studies. The increased risk of miscarriage was found predominantly among those used azithromycin and clarithromycin in pregnancy, but not those used erythromycin (1895 in total). Reasons for this difference are not clear. Fewer gastrointestinal side-effects and better pharmacokinetic profiles (e.g. better oral bioavailability, tissue penetration and longer half-life of elimination) of clarithromycin and azithromycin may increase their maternal and fetal bioavailability compared with erythromycin [48]. Meanwhile, an *ex vivo* experiment with term human placentas found that the transplacental transfer is higher for clarithromycin while similar between azithromycin and erythromycin (percentage transfer at 2.6%, 3% and 6%, respectively) [49].

Timing of treatment according to critical periods of gestation also contributes to heterogeneous effects. Fetuses are most vulnerable to teratogenic effects during certain time windows (e.g., day 28–56 of gestation is the critical period for heart formation) [50, 51]. Romoren et al. found when the window of macrolides exposure was reduced from the first trimester to 28–56 days of gestation, the adjusted OR of cardiovascular malformation increased from 0·96 to 1·36, although they were nonsignificant due to limited power [13]. This increased OR was similar to those reported by Kallen et al in 2005 and 2014[4, 9], which was also consistent with the effect of *I*_Kr_-blockers observed in animal studies and epidemiological studies [50, 52, 53]. These findings highlight the possibility of underestimating the adverse effect of macrolides, by measuring the macrolides exposure outside of critical period for fetal development [2, 27, 36, 54].

### Clinical relevance

According to British National Formula, comment given to erythromycin usage during pregnancy states “Not known to be harmful” [20]. The National Institute for Health and Care Excellence (NICE) guidance recommends erythromycin and clarithromycin as replacements for penicillin allergy for upper respiratory infection (in pregnant women) [55]. The UK Teratology Information Advisory Service recommend that “as the number of documented exposures during pregnancy is limited, azithromycin should be avoided during pregnancy unless alternatives such as erythromycin are inappropriate” and “It would be reasonable, where alternative antimicrobials are available, to avoid clarithromycin in the first trimester of pregnancy” [56–58]. In view of the consistent evidence for an increased risk of miscarriage in mothers prescribed macrolides, with a number needed to harm of 10 in first 6 weeks to 196 at 20 weeks of gestation, guidelines should be updated to avoid macrolides during the first half of pregnancy. The uncertain increased risks of cerebral palsy and epilepsy following macrolide treatment at any point during pregnancy should be reported in drug safety leaflets and alternative antibiotics recommended where appropriate.

### Conclusions

Evidence is consistent with an increased risk of miscarriage in mothers prescribed macrolides during pregnancy compared with alternative antibiotics. The risk of cerebral palsy and epilepsy is uncertain, and there is weak or no evidence for adverse effects on congenital malformations. These findings warrant caution about the use of macrolides in pregnancy and use of alternatives where appropriate. As macrolides are the third most commonly used class of antibiotics, it is important to confirm these results with larger, high quality studies to investigate associations between specific types of macrolides and rare events, such as cerebral palsy, epilepsy and organ-specific malformations.

## Supporting information

S1 Text Review of mechanism studies. (DOCX)

S2 Text Simulation on the effect of survival bias. (DOCX)

S1 Table PRISMA checklist. (DOCX)

S2 Table Search terms. (DOCX)

S3 Table Decision tree for including studies. (DOCX)

S4 Table Risk of bias assessment for randomised controlled trials. (DOCX)

S5 Table Risk of bias assessment for observational studies. (DOCX)

S6 Table Characteristics of included studies. (DOCX)

S1 Fig A suggested pathways from macrolides exposure during pregnancy to adverse child outcomes: through an induced short-term fetal hypoxia. (DOCX)

S2 Fig Primary analysis (observational studies) for the association between adverse child outcomes and prenatal use of macrolides versus alternative antibiotics. (DOCX)

S3 Fig Subgroup analysis: Pooled results for specific macrolide types (observational studies). (DOCX)

S4 Fig Primary analysis (RCTs) for the association between adverse child outcomes and prenatal use of macrolides versus alternative antibiotics. (DOCX)

S5 Fig Secondary analysis (RCTs) for the association between adverse child outcomes and prenatal use of macrolides versus no macrolides. (DOCX)

S6 Fig Subgroup analysis: pooled results for specific macrolide types (RCTs, macrolides versus no macrolides). (DOCX)

S7 Fig Sensitivity analysis according to risk of bias (based on primary analysis). (DOCX)
