# Supplementary material for: Associations between use of macrolide antibiotics during pregnancy and adverse child outcomes: A systematic review and meta-analysis
Source: PLoS One. 2019 Feb 19;14(2):e0212212. doi: 10.1371/journal.pone.0212212 (PMC6380581; doi:10.1371/journal.pone.0212212)
Supplement: S1 Text — (DOCX) [file pone.0212212.s001.docx]

**S1 Text. Review of mechanism studies.**

Warnings have been issued against use of azithromycin and clarithromycin in adults with high risk of cardiovascular complications in the in the United States (US),^1,2^ based on evidence of an unexpected increase in the risk of death and cardiovascular events including arrhythmias and cardiac mortality (Azithromycin: 2 systematic reviews including 20 RCTs;^3,4^ clarithromycin: 1 RCTs^5^).

The *I*_Kr_ channel blocking potential of macrolides first caught attention when cardiac adverse outcomes of macrolides were observed in adults. This *I*_Kr_ channel plays a major role in human cardiac rhythm regulation, in both human adult and fetal cardiomyocytes. Blockage of the *I*_Kr_ channel prolongs the QT interval, which increases the risk of arrhythmic cardiac events.^6-9^ The *I*_Kr_ channel presents in human embryonic cardiomyocytes when the heart starts to beat during 4-5 weeks of gestation.^7,10^ *In vitro* studies have demonstrated that macrolides can block the *I*_Kr_ channel both in human and animal embryonic cells. Macrolides can inhibit CYP450 enzymes and the proarrhythmic properties of macrolides may be exacerbated by their interaction with other CYP450 metabolised QT-prolonging drugs.^6^ (S2 Fig)

Based on limited experimental animal studies data from the manufactures, erythromycin and azithromycin revealed no impairment of fertility or harm to the fetus with daily doses up to maternal toxic levels.^11,12^ Clarithromycin, however, produces fetal death, fetal growth restriction in rabbits and monkeys, and cardiovascular anomalies in rats at low-order multiples of the human dose level.^13^ An *in vitro* study on rat embryos also reported the addition of azithromycin and clarithromycin decreased all growth and developmental parameters in a dose dependent manner.^14^ Based on an *ex vivo* experiment with term human placentas, azithromycin, clarithromycin and erythromycin show transplacental transfer of 2.6%, 3% and 6%, respectively.^15^ Fewer gastrointestinal side-effects and better pharmacokinetic profiles (e.g. better oral bioavailability, tissue penetration and longer half-life of elimination) of clarithromycin and azithromycin may increase their maternal and fetal bioavailability compared with erythromycin.^16^

For other *I*_Kr_ -blockers (e.g. dofetilide, almokalant, and sotalol), teratology studies in rats and rabbits have demonstrated that embryonic or fetal exposure can lead to embryonic death and a wide range of malformations, including digital defects, orofacial clefts and cardiovascular defects, especially ventricular-septal defects^17,18^ Observational studies of mothers prescribed other *I*_Kr_-blocking drugs, e.g. antidepressants citalopram, clomipramine, fluoxetine and paroxetine, report an increased risk of fetal adverse effects including cardiac-septal defects and miscarriages. ^19-22^

Animal studies from different research groups have suggested the mechanism underlying the association between *I*_Kr_ blockers and the observed adverse effects may be embryonic cardiac arrhythmia^23-26^, which results in interrupted or decreased oxygen supply at critical times during fetal development, followed by hypoxia-reoxygenation damage.^27-31^ A dose-response relationship between astemizole (an *I*_Kr_ -blocker) treatment and decrease in heart rate has been observed in rat embryos (indicated by immunohistochemistry).^27^ Additionally, indirect evidence from animal studies suggests that similar malformations to those associated with *I*_Kr_ -blockers can be induced by interrupted or decreased oxygen supply during the embryonic period.^17,32,33^

This evidence from animal studies and observational studies demonstrates that the adverse fetal effects of selected *I*_Kr_ blockers are through hypoxic pathways. In line with this, we hypothesise that fetal exposure to macrolides, as *I*_Kr_ blockers, can also induce short-term periodic hypoxia and result in hypoxic adverse effects in the offspring.

**Reference**

1. FDA U. FDA review finds additional data supports the potential for increased long-term risks with antibiotic clarithromycin (Biaxin) in patients with heart disease. 2018.

2. FDA U. FDA Drug Safety Communication: Azithromycin (Zithromax or Zmax) and the risk of potentially fatal heart rhythms. 2013. https://www.fda.gov/Drugs/DrugSafety/ucm341822.htm (accessed 26.06.2017 2017).

3. Wong AYS, Chan EW, Anand S, Worsley AJ, Wong ICK. Managing Cardiovascular Risk of Macrolides: Systematic Review and Meta-Analysis. Drug safety 2017; 40(8): 663-77.

4. Cheng YJ, Nie XY, Chen XM, et al. The Role of Macrolide Antibiotics in Increasing Cardiovascular Risk. J Am Coll Cardiol 2015; 66(20): 2173-84.

5. Winkel P, Hilden J, Hansen JF, et al. Clarithromycin for stable coronary heart disease increases all-cause and cardiovascular mortality and cerebrovascular morbidity over 10years in the CLARICOR randomised, blinded clinical trial. (1874-1754 (Electronic)).

6. Walter A. Volberg BJK, Weiguo Su, Jing Lin, Jun Zhou. Blockade of Human Cardiac Potassium Channel Human Ether-a-go-go-Related Gene (HERG) by Macrolide Antibiotics. Journal of Pharmacology and Experimental Therapeutics 2002; 302(1): 320-7.

7. Stanat SJ, Carlton CG, Crumb WJ, Jr., Agrawal KC, Clarkson CW. Characterization of the inhibitory effects of erythromycin and clarithromycin on the HERG potassium channel. Molecular and cellular biochemistry 2003; 254(1-2): 1-7.

8. Danielsson C, Brask J, Skold AC, et al. Exploration of human, rat, and rabbit embryonic cardiomyocytes suggests K-channel block as a common teratogenic mechanism. Cardiovascular research 2013; 97(1): 23-32.

9. Skold AC, Danielsson BR. Developmental toxicity in the pregnant rabbit by the class III antiarrhythmic drug sotalol. Pharmacology & toxicology 2001; 88(1): 34-9.

10. Moorman AW, S; Brown, NA; Lamers, W; Anderson, RH. Development of the heart: (1) formation of the cardiac chambers and arterial trunks. Heart (British Cardiac Society) 2003; 89(7): 806-14.

11. Product information. Zithromax. . Pfizer Labs, 1994.

12. Product information. Ery-Tab. Abbott Laboratories, 2000.

13. Product information. Biaxin. Abbott Laboratories, 1996

14. Investigation of developmental toxicity and teratogenicity of macrolide antibiotics in cultured rat embryos. Anat Histol Embryol. 2008 Oct;37(5):369-75.

15. Witt A, Sommer EM, Cichna M, et al. Placental passage of clarithromycin surpasses other macrolide antibiotics. American Journal of Obstetrics and Gynecology 2003; 188(3): 816-9.

16. McKenna S, Evans G, Committee tCIDSAA. Macrolides: A Canadian Infectious Disease Society position paper. Can J Infect Dis 2001; 12(4): 218-31.

17. Danielsson BRA-CSA, F. Class III Antiarrhythmics and Phenytoin: Teratogenicity Due to Embryonic Cardiac Dysrhythmia and Reoxygenation Damage. Current pharmaceutical design 2001; 7: 787-802.

18. Webster WS, Abela D. The effect of hypoxia in development. Birth Defects Res C Embryo Today 2007; 81(3): 215-28.

19. Pedersen LH, Henriksen TB, Vestergaard M, Olsen J, Bech BH. Selective serotonin reuptake inhibitors in pregnancy and congenital malformations: population based cohort study. BMJ 2009; 339: b3569.

20. Kallen B, Otterblad Olausson P. Antidepressant drugs during pregnancy and infant congenital heart defect. Reproductive toxicology (Elmsford, NY) 2006; 21(3): 221-2.

21. Malm H, Artama M, Gissler M, Ritvanen A. Selective serotonin reuptake inhibitors and risk for major congenital anomalies. Obstet Gynecol 2011; 118(1): 111-20.

22. Wurst KE, Poole C, Ephross SA, Olshan AF. First trimester paroxetine use and the prevalence of congenital, specifically cardiac, defects: a meta-analysis of epidemiological studies. Birth defects research Part A, Clinical and molecular teratology 2010; 88(3): 159-70.

23. W. S. Webster PDCB-W, M. D. Snow, B. R. G. Danielsson. Teratogenic potential of almokalant, dofetilide, and d-sotalol: drugs with potassium channel blocking activity. Teratology 1996; 53(3): 168–75.

24. Sköld AC DC, Linder B, Danielsson BR. Teratogenicity of the IKr-blocker cisapride: relation to embryonic cardiac arrhythmia. Reproductive Toxicology 2002; 16(4): 333-42.

25. Ban Y, Konishi R, Kawana K, Nakatsuka T, Fujii T, Manson JM. Embryotoxic effects of L-691,121, a class III antiarrhythmic agent, in rats. Archives of toxicology 1994; 69(1): 65-71.

26. Spence SG, Vetter C, Hoe CM. Effects of the class III antiarrhythmic, dofetilide (UK-68,798) on the heart rate of midgestation rat embryos, in vitro. Teratology 1994; 49(4): 282-92.

27. Nilsson MF, Danielsson C, Skold AC, et al. Improved methodology for identifying the teratogenic potential in early drug development of hERG channel blocking drugs. Reproductive toxicology (Elmsford, NY) 2010; 29(2): 156-63.

28. Danielsson BR, Johansson A, Danielsson C, Azarbayjani F, Blomgren B, Skold AC. Phenytoin teratogenicity: hypoxia marker and effects on embryonic heart rhythm suggest an hERG-related mechanism. Birth defects research Part A, Clinical and molecular teratology 2005; 73(3): 146-53.

29. Bengt R Danielsson A-CS, Alf Johansson, Birgitta Dillner, Bo Blomgren. Teratogenicity by the hERG potassium channel blocking drug almokalant: use of hypoxia marker gives evidence for a hypoxia-related mechanism mediated via embryonic arrhythmia. Toxicology and applied pharmacology 2003; 193(2): 168-76.

30. Patterson AJ, Zhang L. Hypoxia and fetal heart development. Current molecular medicine 2010; 10(7): 653-66.

31. Wellfelt K, Skold AC, Wallin A, Danielsson BR. Teratogenicity of the class III antiarrhythmic drug almokalant. Role of hypoxia and reactive oxygen species. Reproductive toxicology (Elmsford, NY) 1999; 13(2): 93-101.

32. Brent RL, Franklin JB. Uterine vascular clamping: new procedure for the study of congenital malformations. Science (New York, NY) 1960; 132(3419): 89-91.

33. Leist KH, Grauwiler J. Fetal pathology in rats following uterine-vessel clamping on day 14 of gestation. Teratology 1974; 10(1): 55-67.
