# Supplementary material for: Associations between use of macrolide antibiotics during pregnancy and adverse child outcomes: A systematic review and meta-analysis
Source: PLoS One. 2019 Feb 19;14(2):e0212212. doi: 10.1371/journal.pone.0212212 (PMC6380581; doi:10.1371/journal.pone.0212212)
Supplement: S2 Text — (DOCX) [file pone.0212212.s002.docx]

**S2 Text. Simulation on the effect of survival bias.**

This simulation was performed assuming a study on the association between congenital malformation and macrolide antibiotics exposure during pregnancy (versus penicillins). Ideally, results of all pregnancies would be observed as shown in table 10.1.

**Table 10.1 Numbers of subjects observed in all pregnancies.**

| Number of subjects | With malformation | Without malformation |
| --- | --- | --- |
| Macrolides | a | b |
| Penicillins | c | d |

Odds Ratio (in all pregnancies) = ad/bc.

However, both malformation and macrolides were associated with an increased risk of fetal death. Risks of fetal death are estimated as following and in table 10.2:

- The risk of miscarriage in fetuses exposed to penicillin is estimated at 6.7% based on previous observational studies.^1,1^
- Compared to penicillin antibiotics, the risk of fetal death in macrolides group is increased by 50% (about 10.1%, Andersen et al.^1^).
- The risk of fetal death in fetuses with malformation is about 4.5 times of that in unselected population (risk of stillbirth is 0.43% in unselected fetuses and 2% in fetuses with malformation).^3,4^ For simplicity, we also estimated that within fetuses exposed to macrolides, the risk of fetal death would also increase by 4.5 times in fetus with malformation.

**Table 10.2 Risk of fetal death in all pregnancies**.

|  | With malformation | Without malformation |
| --- | --- | --- |
| Macrolides | 45.5% | 10.1% |
| Penicillins | 30.2% | 6.7% |

Thereby the proportions of livebirths in all pregnancies (i.e. those would be selected into future study) are:

**Table 10.3 Proportion of livebirths in all pregnancies.**

|  | With malformation | Without malformation |
| --- | --- | --- |
| Macrolides | 54.5% | 89.9% |
| Penicillins | 69.8% | 93.3% |

Odds Ratio (in livebirths) = (54.5*93.3/(89.9*69.8)) *(ad/bc) =0.81* Odds Ratio (in all pregnancies)

I.e. the OR measured only in livebirths is underestimated given the estimations.

**Reference**

1. Andersen JT, Petersen M, Jimenez-Solem E, et al. Clarithromycin in early pregnancy and the risk of miscarriage and malformation: a register based nationwide cohort study. PLoS One 2013; 8(1): e53327.

2. Einarson A, Phillips E, Mawji F, et al. A prospective controlled multicentre study of clarithromycin in pregnancy. Am J Perinatol 1998; 15(9): 523-5.

3. Statistics OfN. Births in England and Wales: 2016. 2017. https://www.ons.gov.uk/peoplepopulationandcommunity/birthsdeathsandmarriages/livebirths/bulletins/birthsummarytablesenglandandwales/2016.

4. Anne-Marie Nybo Andersen JW, Peter Christens, Jørn Olsen, Mads Melbye. Maternal age and fetal loss: population based register linkage study. British medical journal 2000; 320: 1708-12.
