# Supplementary material for: Associations between use of macrolide antibiotics during pregnancy and adverse child outcomes: A systematic review and meta-analysis
Source: PLoS One. 2019 Feb 19;14(2):e0212212. doi: 10.1371/journal.pone.0212212 (PMC6380581; doi:10.1371/journal.pone.0212212)
Supplement: S2 Table — (DOCX) [file pone.0212212.s004.docx]

**S2 Table. Search terms.**

| Database | Search strategy |
| --- | --- |
| PubMed | #1 Search (Pregnancy[MeSH Terms]) OR Infant[MeSH Terms]  #2 Search (pregnan*[Title/Abstract] OR fetal[Title/Abstract] OR fetus[Title/Abstract] OR maternal[Title/Abstract] OR prenatal[Title/Abstract] OR gestation*[Title/Abstract] OR in utero'[Title/Abstract] OR neona*[Title/Abstract] OR infant*[Title/Abstract] OR infanc*[Title/Abstract])  #3 Search (#1) OR #2  #4 Search macrolide[MeSH Terms]  #5 Search (macrolide*[Title/Abstract] OR erythromycin[Title/Abstract] OR clarithromycin[Title/Abstract] OR azithromycin[Title/Abstract])  #6 Search (#4) OR #5  #7 Search (#3) AND #6  #8 Search (("Clinical Trial"[Publication Type] OR groups[Title/Abstract] OR placebo*[Title/Abstract] OR trial*[Title/Abstract] OR random*[Title/Abstract] OR "drug therapy"[Subheading]) NOT ((animals[MeSH Terms]) NOT ((animals[MeSH Terms]) AND (humans[MeSH Terms]))))  #9 Search (#7) AND #8  #10 Search (penicillins[MeSH Terms]) OR cephalosporins[MeSH Terms]  #11 Search (Penicillin[Text Word] OR Benzylpenicillin[Text Word] OR Phenoxymethylpenicillin[Text Word] OR Crystapen[Text Word] OR Aminopenicillins[Text Word] OR Ampicillin[Text Word] OR Amoxicillin[Text Word] OR Co-amoxiclav[Text Word] OR Amoxil[Text Word] OR Penbritin[Text Word] OR Augmentin[Text Word] OR Flucloxacillin[Text Word] OR CO-FLUAMPICIL[Text Word] OR Magnapen[Text Word] OR Ticarcillin[Text Word] OR Timentin[Text Word] OR PIVMECILLINAM HYDROCHLORIDE[Text Word] OR Selexid[Text Word] OR cephalosporins[Text Word] OR Cefradine[Text Word] OR cefotaxime[Text Word] OR ceftazidime[Text Word] OR cefuroxime[Text Word] OR cefalexin[Text Word] OR ceftriaxone[Text Word] OR CEFACLOR[Text Word] OR Distaclor [Text Word] OR CEFADROXIL[Text Word] OR Baxan[Text Word] OR CEFALEXIN[Text Word] OR Ceporex[Text Word] OR Keflex[Text Word] OR CEFIXIME[Text Word] OR Suprax[Text Word] OR CEFOTAXIME[Text Word] OR CEFPODOXIME[Text Word] OR Orelox [Text Word] OR Velosef[Text Word] OR Fortum[Text Word] OR Kefadim[Text Word] OR Rocephin[Text Word] OR Zinacef[Text Word] OR Zinnat[Text Word])  #12 Search (#10) OR #11  #13 Search (#7) AND #12  #14 Search (#9) OR #13 |
| Cochrane Library | Searched for trials using Cochrane Search Manager:  ID Search Hits  #1 MeSH descriptor: [Pregnancy] explode all trees  #2 MeSH descriptor: [Infant] explode all trees  #3 MeSH descriptor: [Macrolides] explode all trees  #4 #1 or #2  #5 #4 and #3  #6 macrolide or macrolides or erythromycin or clarithromycin or azithromycin :ti,ab,kw and pregnan* or fetal or fetus or maternal or prenatal or gestation* or ‘in utero’ or neona* or infant* or infanc*:ti,ab,kw in Trials (Word variations have been searched)  #7 #6 or #5 |
| Embase | 1. exp pregnancy/  2. exp infant/  3. 1 or 2  4. exp macrolide/  5. 3 and 4  6. exp penicillin derivative/  7. exp cephalosporin derivative/  8. (Penicillin or Benzylpenicillin or Phenoxymethylpenicillin or Crystapen or Aminopenicillins or Ampicillin or Amoxicillin or Co-amoxiclav or Amoxil or Penbritin or Augmentin or Flucloxacillin or CO-FLUAMPICIL or Magnapen or Ticarcillin or Timentin or PIVMECILLINAM HYDROCHLORIDE or Selexid).tw.  9. (cephalosporins or Cefradine or cefotaxime or ceftazidime or cefuroxime or cefalexin or ceftriaxone or CEFACLOR or Distaclor or CEFADROXIL or Baxan or CEFALEXIN or Ceporex or Keflex or CEFIXIME or Suprax or CEFOTAXIME or CEFPODOXIME or Orelox or Velosef or Fortum or Kefadim or Rocephin or Zinacef or Zinnat).tw.  10. 6 or 7 or 8 or 9  11. 5 and 10  12. random*.ab,ti. or placebo*.de,ab,ti. or (double adj1 blind*).ab,ti.  13. 5 and 12  14. 11 or 13  15. limit 14 to human |
| Conference Proceeding Citation Index-Science through Web of Science | TOPIC:(macrolide or macrolides or erythromycin or clarithromycin or azithromycin) AND TOPIC: (pregnant or pregnancy or pregnancies or fetal or maternal or prenatal or gestation or gestational or in utero or neona* or infant* or infanc*)  Timespan: All years. Indexes: CPCI-S. |
| ClinicalTrials.gov | "macrolides" OR "macrolide" OR "erythromycin" OR "clarithromycin" OR "azithromycin"\| Completed Studies \| Studies With Results |
