# Supplementary material for: Associations between use of macrolide antibiotics during pregnancy and adverse child outcomes: A systematic review and meta-analysis
Source: PLoS One. 2019 Feb 19;14(2):e0212212. doi: 10.1371/journal.pone.0212212 (PMC6380581; doi:10.1371/journal.pone.0212212)
Supplement: S3 Table — (DOCX) [file pone.0212212.s005.docx]

**S3 Table. Decision tree for including studies.**

| **Decision tree for including studies** | | | | |
| --- | --- | --- | --- | --- |
|  | **Title and Abstract Review** | **Yes** | **No** | **Maybe** |
| 1 | Does the paper present a trial, a cohort study or a case-control study? | 2 | E | 2 |
| 2 | Does the paper focus on interventions administered during (human) pregnancy? | 3 | E | 3 |
| 3 | Does one of the interventions include macrolide antibiotic (erythromycin, clarithromycin, azithromycin or macrolide antibiotics as a whole)? | 4 | E | 4 |
| 4 | Does the paper present data on offspring outcomes? | Full text | E | Full text |
|  | | | | |
|  | **Full text review** | **Yes** | **No** |  |
| 5 | Does the study present a trial? | 6 | 7 |  |
| 6 | In this trial, does any of the comparison pair belong to any of the following types:  1) macrolide antibiotics VS placebo; 2) macrolide antibiotics VS (penicillins or cefalosporins);  3) (macrolide antibiotics + (penicillins or cefalosporins)) VS (penicillins or cefalosporins) | 10 | E |  |
| 7 | In this observational study, does the comparison belong to penicillins or cefalosporins, or does the data allow a comparison between macrolide antibiotic and penicillins or cefalosporins? | 8 | E |  |
| 8 | Does the study present a case-control study? | 9 | 10 |  |
| 9 | In this case-control study, were the cases and controls selected from a similarly defined population and of which prescription information was collected prospectively? | 10 | E |  |
| 10 | Does the study report the risk (or relevant data to compute the risk) of fetus or child conditions that are direct and specific short-term hypoxia-related effects? | Included | E |  |
