# Supplementary material for: Associations between use of macrolide antibiotics during pregnancy and adverse child outcomes: A systematic review and meta-analysis
Source: PLoS One. 2019 Feb 19;14(2):e0212212. doi: 10.1371/journal.pone.0212212 (PMC6380581; doi:10.1371/journal.pone.0212212)
Supplement: S4 Table — (DOCX) [file pone.0212212.s006.docx]

**S4 Table. Risk of bias assessment for randomised controlled trials.**

| **Bias Domain** | **Source of bias** | **Judgment** | **Support for judgement** |
| --- | --- | --- | --- |
| Selection Bias | Random sequence generation | Low risk | The investigators describe a random component in the sequence generation process for pregnant women e.g. random number table, computer random number generator or shuffling cards or envelopes etc. |
|  |  | High risk | The investigators describe a non-random component in the sequence generation process for pregnant women, e.g. date of birth, day of visit, ID, choice of clinician or participant, test results, availability; or there was insufficient information about the sequence generation process |
|  | Allocation concealment | Low risk | Participants and investigators enrolling participants could not foresee assignment because one of the following, or an equivalent method, was used to conceal allocation:  • Central allocation (including telephone, web-based and pharmacy-controlled randomization);  • Sequentially numbered drug containers of identical appearance;  • Sequentially numbered, opaque, sealed envelopes. |
|  |  | High risk | Participants or investigators enrolling participants could possibly foresee assignments and thus introduce selection bias, such as allocation based on: • Using an open random allocation schedule (e.g. a list of random numbers); • Assignment envelopes were used without appropriate safeguards (e.g. if envelopes were unsealed or nonopaque or not sequentially numbered); • Alternation or rotation; • Date of birth; • Case record number; • Any other explicitly unconcealed procedure. Or there was insufficient information to permit judgement |
| Performance Bias | Blinding of participants and personnel | Low risk | Any one of the following: • Blinding of participants and key study personnel ensured, and unlikely that the blinding could have been broken;  • No blinding or incomplete blinding, but the review authors judge that the outcome is not likely to be influenced by lack of blinding. |
|  |  | High risk | Any one of the following: • No blinding or incomplete blinding, and the outcome is likely to be influenced by lack of blinding; • Blinding of key study participants and personnel attempted, but likely that the blinding could have been broken, and the outcome is likely to be influenced by lack of blinding. Or there was insufficient information to permit judgement. |
| Detection Bias | Blinding of outcome assessment | Low risk | Any one of the following: • Blinding of outcome assessment ensured, and unlikely that the blinding could have been broken;  • No blinding of outcome assessment, but the review authors judge that the outcome measurement is not likely to be influenced by lack of blinding. |
|  |  | High risk | Any one of the following: • No blinding of outcome assessment, and the outcome measurement is likely to be influenced by lack of blinding; • Blinding of outcome assessment, but likely that the blinding could have been broken, and the outcome measurement is likely to be influenced by lack of blinding. Or there was insufficient information to permit judgement |
| Attrition Bias | Incomplete outcome data | Low risk | The follow-up length was long enough to detect specific outcomes. (e.g. at least 1 year to detect birth defects; at least 5 year to detect neurological adverse outcomes), and any one of the following:  •No missing outcome data; •Missing outcome data balanced in numbers across intervention groups, with similar reasons for missing data across groups; •The proportion of missing outcomes compared with observed event risk not enough to have a clinically relevant impact on the intervention effect estimate; •Missing data have been imputed using appropriate methods. |
|  |  | High risk | Any one of the following:  •The follow-up length was not long enough to detect specific outcomes, which would result in a bias towards null; •The proportion of missing outcomes compared with observed event risk enough to induce clinically relevant bias in intervention effect estimate; •‘As-treated’ analysis done with substantial departure of the intervention received from that assigned at randomization; •Potentially inappropriate application of simple imputation. Or there was insufficient information to permit judgement |
| Reporting Bias | Selective reporting | Low risk | Any of the following: •The study protocol is available and all of the study’s pre-specified (primary and secondary) outcomes that are of interest in the review have been reported in the pre-specified way; •The study protocol is not available but it is clear that the published reports include all expected outcomes, including those that were pre-specified (convincing text of this nature may be uncommon). |
|  |  | High risk | Any one of the following: •Not all of the study’s pre-specified primary outcomes have been reported; •One or more primary outcomes is reported using measurements, analysis methods or subsets of the data (e.g. subscales) that were not pre-specified; •One or more reported primary outcomes were not pre-specified (unless clear justification for their reporting is provided, such as an unexpected adverse effect); •One or more outcomes of interest in the review are reported incompletely so that they cannot be entered in a meta-analysis; •The study report fails to include results for a key outcome that would be expected to have been reported for such a study. Or there was insufficient information to permit judgement. |
| Incomparable treatment effect |  | Low risk | •Macrolides were compared with alternative antibiotics. |
|  |  | High risk | RCTs with the following two comparisons:  • macrolides versus placebo;  • macrolides plus alternative antibiotics versus the alternatives. |
