# Supplementary material for: Associations between use of macrolide antibiotics during pregnancy and adverse child outcomes: A systematic review and meta-analysis
Source: PLoS One. 2019 Feb 19;14(2):e0212212. doi: 10.1371/journal.pone.0212212 (PMC6380581; doi:10.1371/journal.pone.0212212)
Supplement: S5 Table — (DOCX) [file pone.0212212.s007.docx]

**S5 Table. Risk of bias assessment for observational studies.**

| **Bias Domain** | **Source of bias** | **Judgment** | **Support for judgement** |
| --- | --- | --- | --- |
| Pre-intervention | Bias due to confounding | Low risk | • Multivariate analysis, propensity score analyses or matching performed with additional confounders (e.g. gestational age, maternal age, and social economic status). |
|  |  | Moderate risk | • The comparison between macrolides and alternative antibiotics was not adjust for additional confounders. |
|  |  | High risk | The risk of bias due to confounding is moderate at worst since we only allow alternative antibiotics as comparator for macrolides, and this comparison itself has controlled for several confounders including infection and social economic status, to a certain degree. |
|  | Bias in selection of participants into the study | Low risk | • In the selection process, there is no other systematic difference introduced between study groups. |
|  |  | High risk | • In cohort studies, the investigator’s selection of exposed and reference groups introduces a systematic difference, other than the exposure, between the groups, and this systematic difference is associated with the outcome. In case-control studies, cases and controls are recruited in a way that they are not representative of the target population. |
| At intervention | Bias in classification of interventions | Low risk | Any one of the following: • The intervention was measured based on prescription records, healthcare registry etc.;  • The intervention was measured before outcome presented. |
|  |  | High risk | Any one of the following: • The intervention was measured after outcome presented;  • The intervention was measured by self-report, which was subject to recall bias.  Or there was insufficient information to permit judgement |
| Post- intervention | Bias due to deviations from intended interventions | Low risk | Observational studies can rarely measure which subjects actually had their prescriptions dispensed and which subjects took antibiotics as dispensed. However, women prescribed macrolides may be less likely to adhere to the treatment than women prescribed penicillin or cephalosporin because of minor side effects of macrolides including nausea, vomiting and diarrhoea, which will result in a bias towards null. |
|  | Bias due to missing | Low risk | • The cohort included methods to document whether the patients were under the follow-up account for censoring, to enable the differentiation between no interested covariates presenting and no information for covariates; or, used person-year to measure the time at risk. And, • For dichotomous outcome data, the proportion of missing outcomes compared with observed event risk is not enough to have an important impact on the intervention effect estimate. |
|  |  | High risk | Any one of the following:  • The cohort did not include methods to document whether the patients were under the follow-up account for censoring, which cannot differentiate between no covariates presenting and no information for covariates;  • For dichotomous outcome data, the proportion of missing outcomes compared with observed event risk is large enough to have an important impact on the intervention effect estimate.  Or there was insufficient information to permit judgement. |
|  | Bias in measurement of outcomes | Low risk | •The follow-up length was long enough to detect specific outcomes. (e.g. at least 1 year to detect birth defects; at least 5 year to detect neurological adverse outcomes), and;  •The cohort included methods to document whether the patients were under the follow-up account for censoring to enable the differentiation between no outcome presenting and no information for outcomes, or used person-year to measure the time at risk. |
|  |  | High risk | Any one of the following:  •The follow-up length was not long enough to detect specific outcomes, which would result in a bias towards null;  •The cohort used proportion instead of person-year measurement, and did not include methods to document whether the patients were under the follow-up account for censoring, which cannot differentiate between no outcome presenting and no information for outcomes; • The outcomes was measured based on self-report.  Or there was insufficient information to permit judgement. |
|  | Bias in selection of the reported results | Low risk | Outcome and analysis reporting were consistent with designing/ pre-specified. |
|  |  | High risk | Selective reporting of a specific outcome (e.g., selected follow-up intervals), incomplete reporting of a specific outcome (e.g., incomplete reporting of nonsignificant p values, such as p>0.05), selective reporting of data on subgroups, presentation of adjusted rather than unadjusted analyses.  Or there was insufficient information to permit judgement. |
