# Supplementary material for: Associations between use of macrolide antibiotics during pregnancy and adverse child outcomes: A systematic review and meta-analysis
Source: PLoS One. 2019 Feb 19;14(2):e0212212. doi: 10.1371/journal.pone.0212212 (PMC6380581; doi:10.1371/journal.pone.0212212)
Supplement: S6 Table — (DOCX) [file pone.0212212.s008.docx]

**S6 Table. Characteristics of included studies.**

| Outcome | Author (Year) (study number). Comparison | Study Design | Maternal risk group | Macrolide type | Timing, length of prescription and follow up^a^ | Comparison type | Macrolides n/N | Comparison n/N | Odds Ratio^b^ | 95% LCL | 95% UCL | Reference |
| --- | --- | --- | --- | --- | --- | --- | --- | --- | --- | --- | --- | --- |
| Miscarriage | Einarson (1998).1 | Prospec cohort | Unselected. All pregnancies of women who inquired about the use of clarithromycin in five centres | Clarithromycin | 4-14 w, NS, NS | Nonteratogenic Antibiotics | 22/157 | 11/166 | 2.30 | 1.07 | 4.91 | 3 |
| Miscarriage | Anderson (2013) | Retro cohort | Unselected. All pregnancies from national fertility registry and Hospital registry | Clarithromycin | 1st tri, NS, NS | Phenoxymethylpenicillin | 40/401 | -/33469 | 1.51^c^ | 1.09 | 2.12 | 7 |
| Miscarriage | Muanda (2017)(1).1 | Nested CC | Unselected. All pregnancies of women who were included in the province's drug insurance plan | Macrolides | Before spontaneous abortion (20w), >=1 prescription, - | Penicilins | 264/1789 | 500/6073 | 1.88^d^ | 1.59 | 2.22 | 8 |
| Miscarriage | Muanda (2017)(1).2 | Nested CC | Same as above | Azithromycin | Same as above | Penicilins | 110/763 | 500/6073 | 1.91^d^ | 1.53 | 2.39 | 8 |
| Miscarriage | Muanda (2017)(1).3 | Nested CC | Same as above | Clarithromycin | Same as above | Penicilins | 111/547 | 500/6073 | 2.73^d^ | 2.16 | 3.44 | 8 |
| Miscarriage | Muanda (2017)(1).4 | Nested CC | Same as above | Erythromycin | Same as above | Penicilins | 29/428 | 500/6073 | 0.82^d^ | 0.56 | 1.19 | 8 |
| Stillbirth | Einarson (1998).2 | Prospec cohort | Unselected. All pregnancies of women who inquired about the use of clarithromycin in five centres | Clarithromycin | 4-14 w, NS, NS | Nonteratogenic Antibiotics | 1/157 | 0/166 | 168.07^e^ | 0.00 | >100 | 3 |
| Stillbirth | Kenyon (2008)(1).1 | RCT | Pprom. All pregnancies of pPROM women (Median gestational age: 32 w) | Erythromycin | 32 w (at randomisation, 61% delivered within 7 days ), 250 mg *4 times a day for 10 days or until delivery, 7 years | Co-amoxiclav | 19/1167 | 22/1180 | 0.87 | 0.47 | 1.62 | 29 |
| Stillbirth | Kenyon (2008)(2).1 | RCT | SPL. All pregnancies of SPL women (Median gestational age: 31 w) | Erythromycin | 31 w (at randomisation, 15.8% delivered within 7 days ), 250 mg *4 times a day for 10 days or until delivery, 7 years | Co-amoxiclav | 9/1204 | 9/1133 | 0.94 | 0.37 | 2.38 | 6 |
| Stillbirth | Eschenbach (1991) | RCT | U. realyticum. All pregnancies of women with U. realyticum (26-30 w ) | Erythromycin | 26-35 w, 333mg 3 times daily, until delivery | Placebo | 3/590 | 3/561 | 0.95 | 0.19 | 4.73 | 28 |
| Stillbirth | McGregor (1991).1 | RCT | pPROM. All pregnancies of pPROM women (30 w) | Erythromycin | 31 w (at randomisation), 333 mg *3 time/day until delivery or 7 days, until delivery | Placebo | 1/28 | 0/27 | 29.04^f^ | 0.00 | >100 | 34 |
| Stillbirth | Mercer (1992) | RCT | pPROM. All pregnancies of pPROM women (20-34 w) | Erythromycin | 20-34 w (at randomisation), 333 mg*3 time/day until delivery, until delivery | Placebo | 2/109 | 5/114 | 0.41 | 0.08 | 2.15 | 35 |
| Stillbirth | Martin (1997) | RCT | Chlamydia trachomatis. All pregnancies of women with Chlamydia trachomatis (29.4 w) | Erythromycin | 23-29 w (at randomisation), 333 mg*3 time/day until 35 w, until delivery | Placebo | 2/202 | 1/203 | 2.02 | 0.18 | 22.46 | 33 |
| Stillbirth | Ye, Y (2001) | RCT | Mycoplasma. All pregnancies of mycoplasma-positive pregnant women ( >=28 w) | Erythromycin | >28 w (at randomisation), 125 mg twice daily for 6 w, until delivery | Placebo | 1/241 | 5/247 | 0.20 | 0.02 | 1.74 | 38 |
| Stillbirth | Kenyon (2008)(1).2 | RCT | pPROM, same as Kenyon (2008)(1).1 | Erythromycin plus co-amoxiclav or erythromycin only | 32 w, same as Kenyon (2008)(1).1 | Co-amoxiclav or placebo | 42/2323 | 44/2389 | 0.98 | 0.64 | 1.50 | 29 |
| Stillbirth | Kenyon (2008)(2).2 | RCT | SPL, same as Kenyon (2008)(2).1 | Erythromycin plus co-amoxiclav or erythromycin only | 31 w, same as Kenyon (2008)(2).1 | Co-amoxiclav or placebo | 20/2375 | 24/2279 | 0.80 | 0.44 | 1.45 | 6 |
| Neonatal death | Kenyon (2001)(1).1 | RCT | pPROM. All pregnancies of pPROM women (Median gestational age: 32 w) | Erythromycin | 32 w (at randomisation, 61% delivered within 7 days ), 250 mg *4 times a day for 10 days or until delivery, until delivery | Co-amoxiclav | 70/1190 | 79/1205 | 0.89 | 0.64 | 1.24 | 31 |
| Neonatal death | Kenyon (2001)(2).1 | RCT | SPL. All pregnancies of SPL women (Median gestational age: 31 w) | Erythromycin | 31 w (at randomisation, 15.8% delivered within 7 days ), 250 mg *4 times a day for 10 days or until delivery, until delivery | Co-amoxiclav | 43/1600 | 38/1534 | 1.09 | 0.70 | 1.69 | 30 |
| Neonatal death | McGregor (1991).2 | RCT | pPROM. All pregnancies of pPROM women (30 w) | Erythromycin | 31 w (at randomisation), 333 mg *3 time/day until delivery or 7 days, until delivery | Placebo | 5/28 | 0/27 | 173.03^f^ | 0.01 | >100 | 34 |
| Neonatal death | Kenyon (2001)(1).2 | RCT | pPROM, same as Kenyon (2001)(1).1 | Erythromycin plus co-amoxiclav or erythromycin only | 32 w, same as Kenyon (2001)(1).1 | Co-amoxiclav or placebo | 147/2397 | 161/2430 | 0.92 | 0.73 | 1.16 | 31 |
| Neonatal death | Kenyon (2001)(2).2 | RCT | SPL, same as Kenyon (2001)(2).1 | Erythromycin plus co-amoxiclav or erythromycin only | 31 w, same as Kenyon (2001)(2).1 | Co-amoxiclav or placebo | 90/3151 | 77/3090 | 1.15 | 0.85 | 1.57 | 30 |
| Neonatal death | Kwak (2013).1 | RCT | All pregnancies of pPROM women (22-23 w) | Clarithromycin + cefazolin | 22-23 w (at randomisation), 250 mg*4 time/day until 35 w, 1 year | Cefazolin | 1/35 | 1/17 | 0.47 | 0.03 | 8.01 | 32 |
| Neonatal death | Kwak (2013).2 | RCT | All pregnancies of pPROM women (22-23 w) | Erythromycin+ cefazolin | 22-23 w (at randomisation), 250 mg*4 time/day until 35 w, 1 year | Cefazolin | 1/31 | 1/17 | 0.53 | 0.03 | 9.11 | 32 |
| Neonatal death | Tita (2016) | RCT | Caesarean delivery. Singletons born to women who had non-elective caesarean delivery during labor or after amniotic membrane rupture (39w) | Azithromycin | 29 w (at randomisation), 500 mg, 3 months | Placebo | 1/1019 | 1/944 | 0.93 | 0.06 | 14.83 | 37 |
| Stillbirth and neonatal mortality | Romoren (2012).1 | Retro cohort | Unselected. All singleton pregnancies. | Macrolides | 1st tri, 90% received 1 course of AB, at least until delivery | Penicilins | 41/5729 | 118/16695 | 1.01 | 0.71 | 1.45 | 13 |
| Cerebral palsy | Kenyon (2008)(1).3 | RCT | pPROM, same as Kenyon (2008)(1).1 | Erythromycin | 32 w, same as Kenyon (2008)(1).1 | Co-amoxiclav | 28/807 | 21/849 | 1.42 | 0.80 | 2.52 | 29 |
| Cerebral palsy | Kenyon (2008)(2).3 | RCT | SPL, same as Kenyon (2008)(2).1 | Erythromycin | 31 w, same as Kenyon (2008)(2).1 | Co-amoxiclav | 18/816 | 15/792 | 1.17 | 0.58 | 2.33 | 6 |
| Cerebral palsy | Meeraus (2015).1 | Retro cohort | Unselected. Term singleton live-births | Macrolides | 1-3 tri, single prescription, until 7 years old (median: 3.6 years) | Penicilins | -/2749 | <55/27577 | 1.43^g^ | 0.66^g^ | 3.06 | 5 |
| Cerebral palsy | Kenyon (2008)(1).4 | RCT | pPROM, same as Kenyon (2008)(1).1 | Erythromycin plus co-amoxiclav or erythromycin only | 32 w, same as Kenyon (2008)(1).1 | Co-amoxiclav or placebo | 46/1590 | 41/1671 | 1.18 | 0.77 | 1.81 | 29 |
| Cerebral palsy | Kenyon (2008)(2).4 | RCT | SPL, same as Kenyon (2008)(2).1 | Erythromycin plus co-amoxiclav or erythromycin only | 31 w, same as Kenyon (2008)(2).1 | Co-amoxiclav or placebo | 53/1611 | 27/1562 | 1.93 | 1.21 | 3.09 | 6 |
| Epilepsy | Kenyon (2008)(1).5 | RCT | pPROM, same as Kenyon (2008)(1).1 | Erythromycin | 32 w, same as Kenyon (2008)(1).1 | Co-amoxiclav | 53/807 | 55/849 | 1.01 | 0.69 | 1.50 | 29 |
| Epilepsy | Kenyon (2008)(2).5 | RCT | SPL, same as Kenyon (2008)(2).1 | Erythromycin | 31 w, same as Kenyon (2008)(2).1 | Co-amoxiclav | 68/816 | 63/792 | 1.05 | 0.74 | 1.50 | 6 |
| Epilepsy | Meeraus (2015).2 | Retro cohort | Unselected. Term singleton live-births | Macrolides | 1-3 tri, single prescription, until 7 years old (median: 3.6 years) | Penicilins | -/2749 | <142/27577 | 2.02^g^ | 1.30^g^ | 3.14 | 5 |
| Epilepsy | Kenyon (2008)(1).6 | RCT | pPROM, same as Kenyon (2008)(1).1 | Erythromycin plus co-amoxiclav or erythromycin only | 32 w, same as Kenyon (2008)(1).1 | Co-amoxiclav or placebo | 101/1590 | 107/1671 | 0.99 | 0.75 | 1.31 | 29 |
| Epilepsy | Kenyon (2008)(2).6 | RCT | SPL, same as Kenyon (2008)(2).1 | Erythromycin plus co-amoxiclav or erythromycin only | 31 w, same as Kenyon (2008)(2).1 | Co-amoxiclav or placebo | 149/1611 | 116/1562 | 1.27 | 0.99 | 1.64 | 6 |
| Cerebral palsy and/or epilepsy | Meeraus (2015).3 | Retro cohort | Unselected. Term singleton live-births | Macrolides | 1-3 tri, single prescription, until 7 years old (median: 3.6 years) | Penicilins | 28/2749 | 156/27577 | 1.78^g^ | 1.18 | 2.69 | 5 |
| Malformation: All | Einarson (1998).3 | Prospec cohort | Unselected. All pregnancies of women who inquired about the use of clarithromycin in five centres | Clarithromycin | 4-14 w, NS, NS | Nonteratogenic Antibiotics | 10/157 | 9/166 | 1.19 | 0.47 | 3.00 | 3 |
| Malformation: All | Cooper (2008).1 | Retro cohort | Unselected. All pregnancies from registry | Erythromycin | 1st tri (0-4 months) , NS , 1 year | Amoxicillin | 23/903 | 232/7216 | 0.79 | 0.51 | 1.21 | 27 |
| Malformation: All | Romoren (2012).2 | Retro cohort | Unselected. All singleton pregnancies. | Macrolides | 1st tri, 90% received 1 course of AB, at least until delivery | Penicilins | 127/2549 | 218/4921 | 1.13 | 0.90 | 1.42 | 13 |
| Malformation: All | Le Nguyen (2017) | Retro cohort | Unselected. All pregnancies of women in the registry French EFEMERIS | Macrolides | 1st tri, NS, NS | Penicilins | 47/2473 | 231/9720 | 0.93^h^ | 0.6 | 1.37 | 10 |
| Malformation: Major | Einarson (1998).4 | Prospec cohort | Unselected. All pregnancies of women who inquired about the use of clarithromycin in five centres | Clarithromycin | 4-14 w, NS, NS | Nonteratogenic Antibiotics | 3/157 | 2/166 | 1.60 | 0.26 | 9.69 | 3 |
| Malformation: Major | Romoren (2012).3 | Retro cohort | Unselected. All singleton pregnancies. | Macrolides | 1st tri, 90% received 1 course of AB, at least until delivery | Penicilins | 69/2549 | 139/4921 | 0.96 | 0.71 | 1.28 | 13 |
| Malformation: Major | Muanda (2017)(2).1 | Retro cohort | Unselected. Live-born singletons of women in the national birth registry Quebec pregnancy cohort | Macrolides | 1st tri, NS, 1 year | Penicilins | 265/2332 | 894/9106 | 1.18 | 1.02 | 1.36 | 36 |
| Malformation: Major | Muanda (2017)(2).10 | Retro cohort | Unselected, see Muanda (2017)(2).1 | Azithromycin | 1st tri, NS, 1 year | Amoxicillin | 118/883 | 584/5950 | 1.42 | 1.15 | 1.75 | 36 |
| Malformation: Major | Muanda (2017)(2).11 | Retro cohort | Unselected, see Muanda (2017)(2).1 | Clarithromycin | 1st tri, NS, 1 year | Amoxicillin | 77/658 | 584/5950 | 1.22 | 0.95 | 1.57 | 36 |
| Malformation: Major | Muanda (2017)(2).12 | Retro cohort | Unselected, see Muanda (2017)(2).1 | Erythromycin | 1st tri, NS, 1 year | Amoxicillin | 64/697 | 584/5950 | 0.93 | 0.71 | 1.22 | 36 |
| Malformation: Nervous System | Cooper (2008).2 | Retro cohort | Unselected. All pregnancies from registry | Erythromycin | 1st tri (0-4 months) , NS , 1 year | Amoxicillin | 1/903 | 23/7216 | 0.35 | 0.05 | 2.57 | 27 |
| Malformation: Nervous System | Muanda (2017)(2).2 | Retro cohort | Unselected, see Muanda (2017)(2).1 | Macrolides | 1st tri, NS, 1 year | Penicilins | 16/2332 | 53/9106 | 1.18 | 0.67 | 2.07 | 36 |
| Malformation: Nervous System | Muanda (2017)(2).13 | Retro cohort | Unselected, see Muanda (2017)(2).1 | Azithromycin | 1st tri, NS, 1 year | Amoxicillin | 8/883 | 33/5950 | 1.64 | 0.75 | 3.56 | 36 |
| Malformation: Nervous System | Muanda (2017)(2).14 | Retro cohort | Unselected, see Muanda (2017)(2).1 | Clarithromycin | 1st tri, NS, 1 year | Amoxicillin | 4/658 | 33/5950 | 1.10 | 0.39 | 3.11 | 36 |
| Malformation: Nervous System | Muanda (2017)(2).15 | Retro cohort | Unselected, see Muanda (2017)(2).1 | Erythromycin | 1st tri, NS, 1 year | Amoxicillin | 2/697 | 33/5950 | 0.52 | 0.12 | 2.15 | 36 |
| Malformation: Orofacial | Cooper (2008).3 | Retro cohort | Unselected. All pregnancies from registry | Erythromycin | 1st tri (0-4 months) , NS , 1 year | Amoxicillin | 0/903 | 15/7216 | 0.00^i^ | 0.00 | >100 | 27 |
| Malformation: Orofacial | Muanda (2017)(2).3 | Retro cohort | Unselected, see Muanda (2017)(2).1 | Macrolides | 1st tri, NS, 1 year | Penicilins | 16/2332 | 51/9106 | 1.23 | 0.70 | 2.15 | 36 |
| Malformation: Orofacial | Muanda (2017)(2).16 | Retro cohort | Unselected, see Muanda (2017)(2).1 | Azithromycin | 1st tri, NS, 1 year | Amoxicillin | 2/883 | 1479354 | 2.70 | 0.52 | 13.93 | 36 |
| Malformation: Orofacial | Muanda (2017)(2).17 | Retro cohort | Unselected, see Muanda (2017)(2).1 | Clarithromycin | 1st tri, NS, 1 year | Amoxicillin | 2/658 | 1479354 | 3.63 | 0.70 | 18.72 | 36 |
| Malformation: Orofacial | Muanda (2017)(2).18 | Retro cohort | Unselected, see Muanda (2017)(2).1 | Erythromycin | 1st tri, NS, 1 year | Amoxicillin | 3/697 | 1479354 | 5.14 | 1.23 | 21.55 | 36 |
| Malformation: Cardiovascular | Kallen (2005).1 | Retro cohort | Unselected. Livebirths of women in the national birth registry | Erythromycin | 1st tri, NS, 1 year | Penicilins | 31/1844 | 84/9110 | 1.84 | 1.21 | 2.78 | 4 |
| Malformation: Cardiovascular | Cooper (2008).4 | Retro cohort | Unselected. All pregnancies from registry | Erythromycin | 1st tri (0-4 months) , NS , 1 year | Amoxicillin | 9/903 | 89/7216 | 0.81 | 0.40 | 1.61 | 27 |
| Malformation: Cardiovascular | Romoren (2012).4 | Retro cohort | Unselected. All singleton pregnancies. | Macrolides | 1st tri, 90% received 1 course of AB, at least until delivery | Penicilins | 25/2549 | 46/4921 | 1.05 | 0.64 | 1.71 | 13 |
| Malformation: Cardiovascular | Muanda (2017)(2).4 | Retro cohort | Unselected, see Muanda (2017)(2).1 | Macrolides | 1st tri, NS, 1 year | Penicilins | 47/2332 | 192/9106 | 0.95 | 0.69 | 1.32 | 36 |
| Malformation: Cardiovascular | Muanda (2017)(2).19 | Retro cohort | Unselected, see Muanda (2017)(2).1 | Azithromycin | 1st tri, NS, 1 year | Amoxicillin | 19/883 | 117/5950 | 1.10 | 0.67 | 1.79 | 36 |
| Malformation: Cardiovascular | Muanda (2017)(2).20 | Retro cohort | Unselected, see Muanda (2017)(2).1 | Clarithromycin | 1st tri, NS, 1 year | Amoxicillin | 12/658 | 117/5950 | 0.93 | 0.51 | 1.69 | 36 |
| Malformation: Cardiovascular | Muanda (2017)(2).21 | Retro cohort | Unselected, see Muanda (2017)(2).1 | Erythromycin | 1st tri, NS, 1 year | Amoxicillin | 15/697 | 117/5950 | 1.10 | 0.64 | 1.89 | 36 |
| Malformation: VSD/ASD | Kallen (2005).2 | Retro cohort | Unselected. Livebirths of women in the national birth registry | Erythromycin | 1st tri, NS, 1 year | Penicilins | 18/1844 | 57/9110 | 1.57 | 0.92 | 2.67 | 4 |
| Malformation: VSD/ASD | Romoren (2012).5 | Retro cohort | Unselected. All singleton pregnancies. | Macrolides | 1st tri, 90% received 1 course of AB, at least until delivery | Penicilins | 19/2549 | 29/4921 | 1.27 | 0.71 | 2.26 | 13 |
| Malformation: VSD/ASD | Muanda (2017)(2).5 | Retro cohort | Unselected, see Muanda (2017)(2).1 | Macrolides | 1st tri, NS, 1 year | Penicilins | 35/2332 | 150/9106 | 0.91 | 0.63 | 1.32 | 36 |
| Malformation: VSD/ASD | Muanda (2017)(2).22 | Retro cohort | Unselected, see Muanda (2017)(2).1 | Azithromycin | 1st tri, NS, 1 year | Amoxicillin | 14/883 | 93/5950 | 1.01 | 0.58 | 1.79 | 36 |
| Malformation: VSD/ASD | Muanda (2017)(2).23 | Retro cohort | Unselected, see Muanda (2017)(2).1 | Clarithromycin | 1st tri, NS, 1 year | Amoxicillin | 9/658 | 93/5950 | 0.87 | 0.44 | 1.74 | 36 |
| Malformation: VSD/ASD | Muanda (2017)(2).24 | Retro cohort | Unselected, see Muanda (2017)(2).1 | Erythromycin | 1st tri, NS, 1 year | Amoxicillin | 11/697 | 93/5950 | 1.01 | 0.54 | 1.90 | 36 |
| Malformation: Respiratory | Muanda (2017)(2).6 | Retro cohort | Unselected, see Muanda (2017)(2).1 | Macrolides | 1st tri, NS, 1 year | Penicilins | 14/2332 | 45/9106 | 1.22 | 0.67 | 2.22 | 36 |
| Malformation: Respiratory | Muanda (2017)(2).25 | Retro cohort | Unselected, see Muanda (2017)(2).1 | Azithromycin | 1st tri, NS, 1 year | Amoxicillin | 6/883 | 30/5950 | 1.35 | 0.56 | 3.25 | 36 |
| Malformation: Respiratory | Muanda (2017)(2).26 | Retro cohort | Unselected, see Muanda (2017)(2).1 | Clarithromycin | 1st tri, NS, 1 year | Amoxicillin | 4/658 | 30/5950 | 1.21 | 0.42 | 3.44 | 36 |
| Malformation: Respiratory | Muanda (2017)(2).27 | Retro cohort | Unselected, see Muanda (2017)(2).1 | Erythromycin | 1st tri, NS, 1 year | Amoxicillin | 4/697 | 30/5950 | 1.14 | 0.40 | 3.24 | 36 |
| Malformation: Gastrointestinal | Cooper (2008).5 | Retro cohort | Unselected. All pregnancies from registry | Erythromycin | 1st tri (0-4 months) , NS , 1 year | Amoxicillin | 0/903 | 26/7216 | 0.00^i^ | 0.00 | >100 | 27 |
| Malformation: Gastrointestinal | Muanda (2017)(2).7 | Retro cohort | Unselected, see Muanda (2017)(2).1 | Macrolides | 1st tri, NS, 1 year | Penicilins | 35/2332 | 88/9106 | 1.56 | 1.05 | 2.32 | 36 |
| Malformation: Gastrointestinal | Muanda (2017)(2).28 | Retro cohort | Unselected, see Muanda (2017)(2).1 | Azithromycin | 1st tri, NS, 1 year | Amoxicillin | 15/883 | 54/5950 | 1.89 | 1.06 | 3.36 | 36 |
| Malformation: Gastrointestinal | Muanda (2017)(2).29 | Retro cohort | Unselected, see Muanda (2017)(2).1 | Clarithromycin | 1st tri, NS, 1 year | Amoxicillin | 10/658 | 54/5950 | 1.68 | 0.85 | 3.32 | 36 |
| Malformation: Gastrointestinal | Muanda (2017)(2).30 | Retro cohort | Unselected, see Muanda (2017)(2).1 | Erythromycin | 1st tri, NS, 1 year | Amoxicillin | 10/697 | 54/5950 | 1.59 | 0.81 | 3.14 | 36 |
| Malformation: Cleft palate/lip | Muanda (2017)(2).8 | Retro cohort | Unselected, see Muanda (2017)(2).1 | Macrolides | 1st tri, NS, 1 year | Penicilins | 157968 | 13/9106 | 2.11 | 0.84 | 5.28 | 36 |
| Malformation: Cleft palate/lip | Muanda (2017)(2).31 | Retro cohort | Unselected, see Muanda (2017)(2).1 | Azithromycin | 1st tri, NS, 1 year | Amoxicillin | 2/883 | 5/5950 | 2.70 | 0.52 | 13.93 | 36 |
| Malformation: Cleft palate/lip | Muanda (2017)(2).32 | Retro cohort | Unselected, see Muanda (2017)(2).1 | Clarithromycin | 1st tri, NS, 1 year | Amoxicillin | 2/658 | 5/5951 | 3.63 | 0.70 | 18.72 | 36 |
| Malformation: Cleft palate/lip | Muanda (2017)(2).33 | Retro cohort | Unselected, see Muanda (2017)(2).1 | Erythromycin | 1st tri, NS, 1 year | Amoxicillin | 3/697 | 5/5952 | 5.14 | 1.23 | 21.55 | 36 |
| Malformation: Pyloric stenosis | Kallen (2005).3 | Retro cohort | Unselected. Live-births of women in the national birth registry | Erythromycin | 1st tri, NS, 1 year | Penicilins | 4/1844 | 6/5953 | 2.47 | 0.62 | 9.90 | 4 |
| Malformation: Pyloric stenosis | Lund (2014).1 | Retro cohort | Unselected. Live-born singletons from Danish national patient register | Macrolides | 1-2 tri, NS, NS | Penicilins | 20/7569 | 89/34222 | 1.02 | 0.63 | 1.65 | 39 |
| Malformation: Pyloric stenosis | Lund (2014).3 | Retro cohort | Unselected. Live-born singletons from Danish national patient register | Azithromycin | 1-2 tri, NS, NS | Penicilins | 7/1574 | 89/34222 | 1.71 | 0.79 | 3.69 | 39 |
| Malformation: Pyloric stenosis | Lund (2014).4 | Retro cohort | Unselected. Live-born singletons from Danish national patient register | Clarithromycin | 1-2 tri, NS, NS | Penicilins | 1/223 | 89/34222 | 1.72 | 0.24 | 12.38 | 39 |
| Malformation: Pyloric stenosis | Lund (2014).5 | Retro cohort | Unselected. Live-born singletons from Danish national patient register | Erythromycin | 1-2 tri, NS, NS | Penicilins | 6/4528 | 89/34222 | 0.51 | 0.22 | 1.16 | 39 |
| Malformation: Pyloric stenosis | Lund (2014).2 | Retro cohort | Unselected. Live-born singletons from Danish national patient register | Macrolides | 1-2 tri, NS, NS | Penicilins | 10/2286 | 40/16655 | 1.82 | 0.91 | 3.64 | 39 |
| Malformation: Genitourinary | Cooper (2008).6 | Retro cohort | Unselected. All pregnancies from registry | Erythromycin | 1st tri (0-4 months) , NS , 1 year | Amoxicillin | 7/903 | 52/7216 | 1.08 | 0.49 | 2.38 | 27 |
| Malformation: Genitourinary | Muanda (2017)(2).9 | Retro cohort | Unselected, see Muanda (2017)(2).1 | Macrolides | 1st tri, NS, 1 year | Penicilins | 41/2332 | 154/9106 | 1.04 | 0.73 | 1.47 | 36 |
| Malformation: Genitourinary | Muanda (2017)(2).34 | Retro cohort | Unselected, see Muanda (2017)(2).1 | Azithromycin | 1st tri, NS, 1 year | Amoxicillin | 21/883 | 102/5950 | 1.40 | 0.87 | 2.25 | 36 |
| Malformation: Genitourinary | Muanda (2017)(2).35 | Retro cohort | Unselected, see Muanda (2017)(2).1 | Clarithromycin | 1st tri, NS, 1 year | Amoxicillin | 9/658 | 102/5950 | 0.80 | 0.40 | 1.58 | 36 |
| Malformation: Genitourinary | Muanda (2017)(2).36 | Retro cohort | Unselected, see Muanda (2017)(2).1 | Erythromycin | 1st tri, NS, 1 year | Amoxicillin | 11/697 | 102/5950 | 0.92 | 0.49 | 1.72 | 36 |
| Malformation: Musculoskeletal | Cooper (2008).7 | Retro cohort | Unselected. All pregnancies from registry | Erythromycin | 1st tri (0-4 months) , NS , 1 year | Amoxicillin | 5/903 | 52/7216 | 0.77 | 0.31 | 1.93 | 27 |
| Malformation: Musculoskeletal | Muanda (2017)(2).10 | Retro cohort | Unselected, see Muanda (2017)(2).1 | Macrolides | 1st tri, NS, 1 year | Penicilins | 103/2332 | 335/9106 | 1.21 | 0.97 | 1.52 | 36 |
| Malformation: Musculoskeletal | Muanda (2017)(2).37 | Retro cohort | Unselected, see Muanda (2017)(2).1 | Azithromycin | 1st tri, NS, 1 year | Amoxicillin | 48/883 | 230/5950 | 1.43 | 1.04 | 1.97 | 36 |
| Malformation: Musculoskeletal | Muanda (2017)(2).38 | Retro cohort | Unselected, see Muanda (2017)(2).1 | Clarithromycin | 1st tri, NS, 1 year | Amoxicillin | 30/658 | 230/5950 | 1.19 | 0.80 | 1.75 | 36 |
| Malformation: Musculoskeletal | Muanda (2017)(2).39 | Retro cohort | Unselected, see Muanda (2017)(2).1 | Erythromycin | 1st tri, NS, 1 year | Amoxicillin | 25/697 | 230/5950 | 0.93 | 0.61 | 1.41 | 36 |
| Malformation: Craniosynostosis | Muanda (2017)(2).11 | Retro cohort | Unselected, see Muanda (2017)(2).1 | Macrolides | 1st tri, NS, 1 year | Penicilins | 15/2332 | 41/9106 | 1.43 | 0.79 | 2.59 | 36 |
| Malformation: Craniosynostosis | Muanda (2017)(2).40 | Retro cohort | Unselected, see Muanda (2017)(2).1 | Azithromycin | 1st tri, NS, 1 year | Amoxicillin | 8/883 | 27/5950 | 2.01 | 0.91 | 4.43 | 36 |
| Malformation: Craniosynostosis | Muanda (2017)(2).41 | Retro cohort | Unselected, see Muanda (2017)(2).1 | Clarithromycin | 1st tri, NS, 1 year | Amoxicillin | 4/658 | 27/5950 | 1.34 | 0.47 | 3.85 | 36 |
| Malformation: Craniosynostosis | Muanda (2017)(2).42 | Retro cohort | Unselected, see Muanda (2017)(2).1 | Erythromycin | 1st tri, NS, 1 year | Amoxicillin | 3/697 | 27/5950 | 0.95 | 0.29 | 3.13 | 36 |

a. Priority of timing was given to median gestation age of exposure or randomisation, followed by mean, range and approximate time window of exposure; w: gestational week. b. Adjusted odds ratio/ hazard ratio was also shown if available. c. In the study of Andersen (2013), OR was adjusted by maternal age, number of previous miscarriages, income and education. Number of miscarriage in comparison group not given. d. In the study of Muanda (2017), cases and controls were matched by gestational age and year of pregnancy; OR was adjusted by 11 covariates, e.g. maternal age, education level, chronic comorbidities, maternal infections (urinary tract infection, respiratory tract infection, bacterial vaginosis and sexually transmitted infections) and prior exposure to antibiotics. e. In the study of Einarson (1998), the count in clarithromycin arm was adjusted by adding the reciprocal of the size of the opposite treatment arm size (1/166) and non-teratogenic antibiotics arm adjusted by adding 1/157, due to zero event. f. In the study of McGregor (1991), the counts in erythromycin arm were adjusted by adding the reciprocal of the size of the opposite treatment arm size (1/27) and placebo arm adjusted by adding 1/28, due to zero event. g. In the study of Meeraus (2015), there was a total of 55 cerebral palsy cases in macrolides group and penicillins group, with specific number in each group not given. There was a total of 142 epilepsy cases in macrolides group and penicillin group, with specific number in each group not given. The HR was adjusted by maternal age, Townsend quintile, year of delivery, smoking/tobacco use, alcohol problems, obesity, illicit drug use, treatment of chronic medical conditions and potentially neurologically-damaging infection during pregnancy. h. In the study of Le guyen, OR was adjusted by maternal age, long-term illnesses, parity and multiple pregnancy. i. In the study of Cooper (2008), the counts in erythromycin arm were adjusted by adding the reciprocal of the size of the opposite treatment arm size (1/7216) and Amoxicillin arm adjusted by adding 1/903, due to zero event. OR: Odds Ratio; 95% LCL: Lower 95% confidence interval; 95% UCL: Upper 95% confidence interval; RCT: Randomised clinical trial; CC: case control; pPROM: Preterm premature rupture of membranes; SPL: spontaneous preterm labour.
