# Supplementary material for: Associations between use of macrolide antibiotics during pregnancy and adverse child outcomes: A systematic review and meta-analysis
Source: PLoS One. 2019 Feb 19;14(2):e0212212. doi: 10.1371/journal.pone.0212212 (PMC6380581; doi:10.1371/journal.pone.0212212)
Supplement: S1 Fig — (DOCX) [file pone.0212212.s009.docx]

**S1 Fig. A suggested pathways from macrolides exposure during pregnancy to adverse child outcomes: through an induced short-term fetal hypoxia.**

Arrhythmia-related cardiac effects of macrolides in adults

IKr blockers

*I*_Kr_ blocking potential of macrolides

Short term fetal hypoxia

Adverse child outcomes

Observed and hypothesised associations

in fetus

Observed association in adults

Arrhythmia-related effects

Solid line: observed association; Dash line: hypothesised association. *I*_Kr_: rapidly activating component of delayed rectifier K current, which conducts potassium (K+) ions out of cardiac myocytes.
