# Supplementary material for: Associations between use of macrolide antibiotics during pregnancy and adverse child outcomes: A systematic review and meta-analysis
Source: PLoS One. 2019 Feb 19;14(2):e0212212. doi: 10.1371/journal.pone.0212212 (PMC6380581; doi:10.1371/journal.pone.0212212)
Supplement: S2 Fig — (DOCX) [file pone.0212212.s010.docx]

**S2 Fig. Primary analysis (observational studies) for the association between adverse child outcomes and prenatal use of macrolides versus alternative antibiotics.**


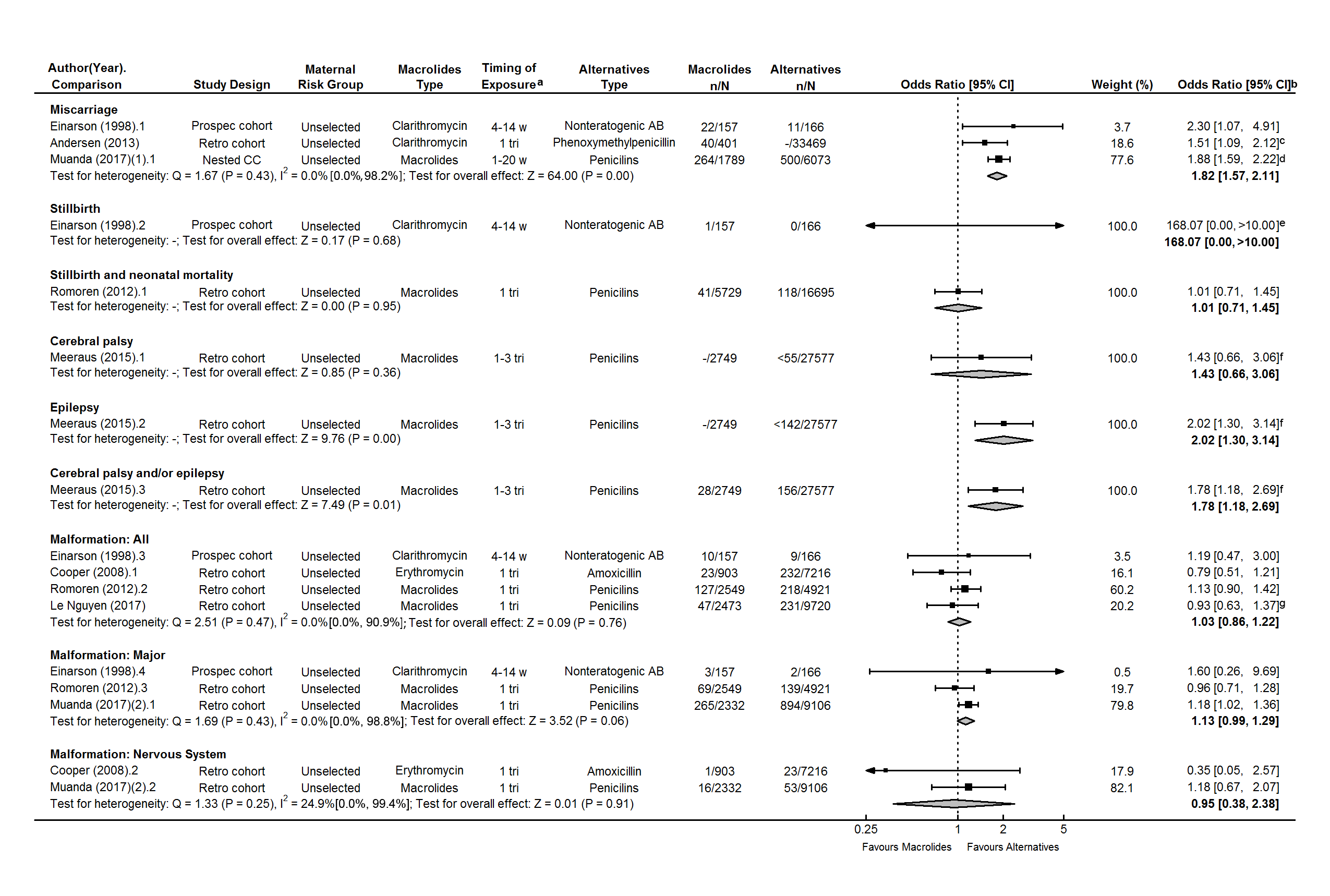


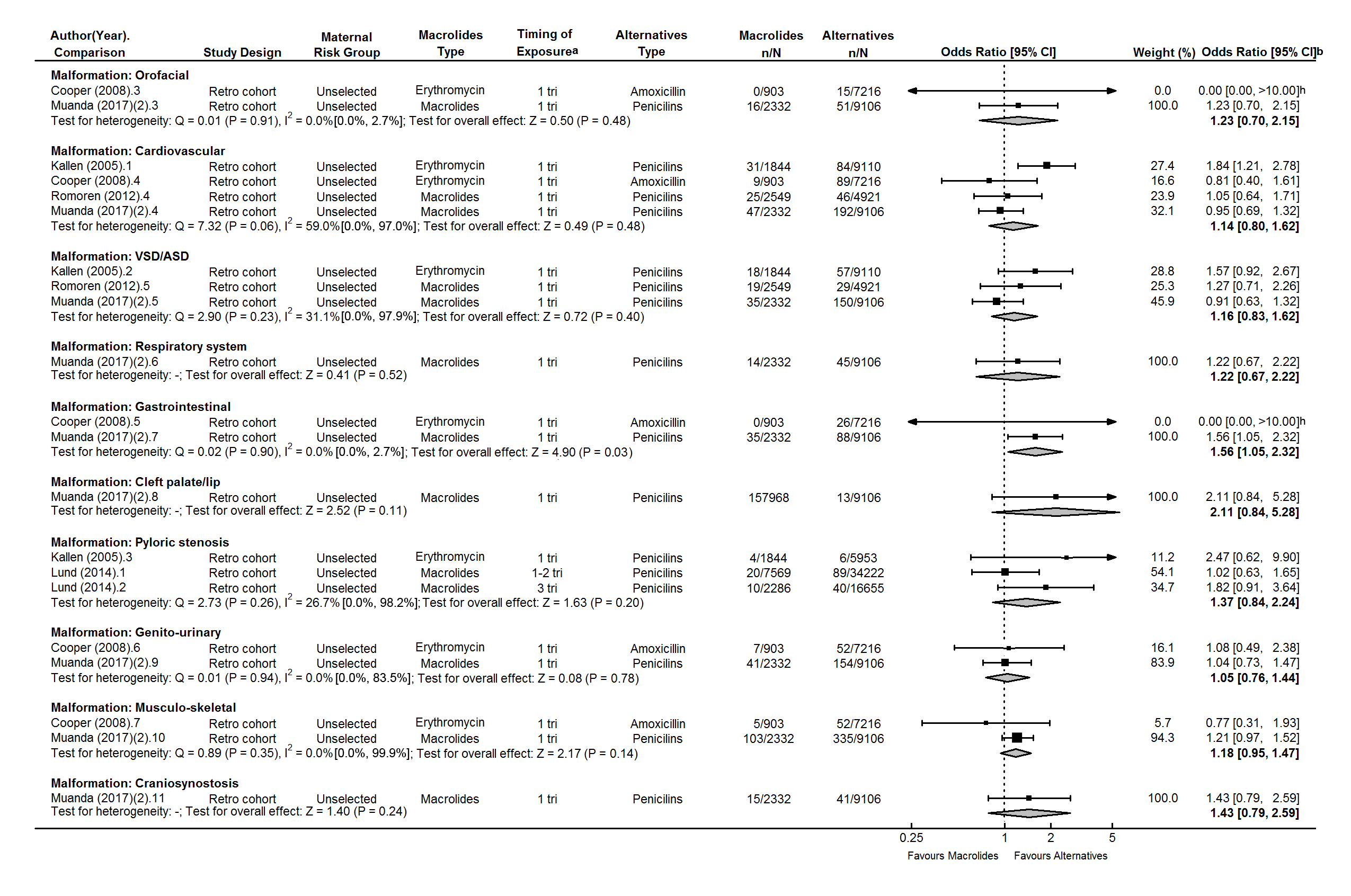
 (S9 Fig continued)

a. Priority of timing was given to median gestation age of exposure or randomisation, followed by mean, range and approximate time window of exposure; w: gestational week. b. Adjusted odds ratio/ hazard ratios were shown if available. c. In the study of Andersen (2013), OR was adjusted by maternal age, number of previous miscarriages, income and education. Number of miscarriage in the comparison group was not given. d. In the study of Muanda (2017), cases and controls were matched by gestational age and year of pregnancy; OR was adjusted by 11 covariates, e.g. maternal age, education level, chronic comorbidities, maternal infections (urinary tract infection, respiratory tract infection, bacterial vaginosis and sexually transmitted infections) and prior exposure to antibiotics. e. In the study of Einarson (1998), the count in clarithromycin arm was adjusted by adding the reciprocal of the size of the opposite treatment arm size (1/166) and non-teratogenic antibiotics arm adjusted by adding 1/157, due to zero event. f. In the study of Meeraus (2015), there were a total of 55 cerebral palsy cases and 142 epilepsy cases in macrolides group and penicillins group, with specific number in each group not given. The hazard ratio was adjusted by maternal age, Townsend quintile, year of delivery, smoking/tobacco use, alcohol problems, obesity, illicit drug use, treatment of chronic medical conditions and potentially neurologically-damaging infection during pregnancy. g. In the study of Le guyen, OR was adjusted by maternal age, long-term illnesses, parity and multiple pregnancy. h. In the study of Cooper (2008), the counts in erythromycin arm were adjusted by adding the reciprocal of the size of the opposite treatment arm size (1/7216) and Amoxicillin arm adjusted by adding 1/903, due to zero event. Prospec: prospective; Retro: retrospective; CC: case control; AB: antibiotics.
