# Supplementary material for: Associations between use of macrolide antibiotics during pregnancy and adverse child outcomes: A systematic review and meta-analysis
Source: PLoS One. 2019 Feb 19;14(2):e0212212. doi: 10.1371/journal.pone.0212212 (PMC6380581; doi:10.1371/journal.pone.0212212)
Supplement: S3 Fig — (DOCX) [file pone.0212212.s011.docx]

**S3 Fig. Subgroup analysis: pooled results for specific macrolide types (observational studies).**

| Outcomes | Subgroup | Number of Studies | Macrolides n/N | Alternatives n/N | I^2^ | Pooled OR  (95% CI) | Pooled OR (95% CI) | Author (year)(paper number), comparison number |
| --- | --- | --- | --- | --- | --- | --- | --- | --- |
| Miscarriage | Azithromycin | 1 | 110/763 | 500/6073 | - | 1.91 [1.53, 2.39] |  | Muanda (2017)(1).2 |
|  | Clarithromycin | 3 | 133/704 | 511/6239 | 74.98 | 2.11 [1.35, 3.30] |  | Muanda (2017)(1).3, Einarson (1998).1, Anderson (2013) |
|  | Erythromycin | 1 | 29/428 | 500/6073 | - | 0.82 [0.57, 1.19] |  | Muanda (2017)(1).4 |
| Stillbirth | Clarithromycin | 1 | 1/157 | 0/166 | - | 168.07 [0, >100.00] |  | Einarson (1998).2 |
| Malformation: All | Clarithromycin | 1 | 10/157 | 9/166 | - | 1.19 [0.47, 3.00] |  | Einarson (1998).3 |
|  | Erythromycin | 1 | 23/903 | 232/7216 | - | 0.79 [0.51, 1.21] |  | Cooper (2008).1 |
| Malformation: Major | Azithromycin | 1 | 118/883 | 584/5950 | - | 1.42 [1.15, 1.75] |  | Muanda (2017)(2).10 |
|  | Clarithromycin | 2 | 80/815 | 586/6116 | 0 | 1.22 [0.95, 1.57] |  | Muanda (2017)(2).11, Einarson (1998).4 |
|  | Erythromycin | 1 | 64/697 | 584/5950 | - | 0.93 [0.71, 1.22] |  | Muanda (2017)(2).12 |
| Malformation: Nervous System | Azithromycin | 1 | 8/883 | 33/5950 | - | 1.64 [0.75, 3.56] |  | Muanda (2017)(2).13 |
|  | Clarithromycin | 1 | 4/658 | 33/5950 | - | 1.10 [0.39, 3.11] |  | Muanda (2017)(2).14 |
|  | Erythromycin | 2 | 3/1600 | 56/13166 | 0 | 0.45 [0.14, 1.44] |  | Muanda (2017)(2).15, Cooper (2008).2 |
| Malformation: Orafacial | Azithromycin | 1 | 2/883 | 5/5950 | - | 2.70 [0.52, 13.93] |  | Muanda (2017)(2).16 |
|  | Clarithromycin | 1 | 2/658 | 5/5950 | - | 3.62 [0.70, 18.72] |  | Muanda (2017)(2).17 |
|  | Erythromycin | 2 | 3/1600 | 20/13166 | 0 | 5.14 [1.22, 21.53] |  | Muanda (2017)(2).18, Cooper (2008).3 |
| Malformation: Cardiovascular | Azithromycin | 1 | 19/883 | 117/5950 | - | 1.10 [0.67, 1.79] |  | Muanda (2017)(2).19 |
|  | Clarithromycin | 1 | 12/658 | 117/5950 | - | 0.93 [0.51, 1.69] |  | Muanda (2017)(2).20 |
|  | Erythromycin | 3 | 55/3444 | 290/22276 | 58.61 | 1.24 [0.77, 2.02] |  | Muanda (2017)(2).21, Kallen (2005).1, Cooper (2008).4 |
| Malformation: Gastrointestinal | Azithromycin | 1 | 15/883 | 54/5950 | - | 1.89 [1.06, 3.36] |  | Muanda (2017)(2).28 |
|  | Clarithromycin | 1 | 10/658 | 54/5950 | - | 1.68 [0.85, 3.32] |  | Muanda (2017)(2).29 |
|  | Erythromycin | 2 | 10/1600 | 80/13166 | 0 | 1.59 [0.81, 3.13] |  | Muanda (2017)(2).30, Cooper (2008).5 |
| Malformation: Genitourinary | Azithromycin | 1 | 21/883 | 102/5950 | - | 1.40 [0.87, 2.25] |  | Muanda (2017)(2).34 |
|  | Clarithromycin | 1 | 9/658 | 102/5950 | - | 0.80 [0.40, 1.58] |  | Muanda (2017)(2).35 |
|  | Erythromycin | 2 | 18/1600 | 154/13166 | 0 | 0.98 [0.60, 1.60] |  | Muanda (2017)(2).36, Cooper (2008).6 |
| Malformation: Musculoskeletal | Azithromycin | 1 | 48/883 | 230/5950 | - | 1.43 [1.04, 1.97] |  | Muanda (2017)(2).37 |
|  | Clarithromycin | 1 | 30/658 | 230/5950 | - | 1.19 [0.80, 1.75] |  | Muanda (2017)(2).38 |
|  | Erythromycin | 2 | 30/1600 | 282/13166 | 0 | 0.90 [0.61, 1.31] |  | Muanda (2017)(2).39, Cooper (2008).7 |
| Malformation: VSD/ASD | Azithromycin | 1 | 14/883 | 93/5950 | - | 1.01 [0.58, 1.79] |  | Muanda (2017)(2).22 |
|  | Clarithromycin | 1 | 9/658 | 93/5950 | - | 0.87 [0.44, 1.74] |  | Muanda (2017)(2).23 |
|  | Erythromycin | 2 | 29/2541 | 150/15060 | 7.91 | 1.30 [0.85, 1.99] |  | Muanda (2017)(2).24, Kallen (2005).2 |
| Malformation: Respiratory system | Azithromycin | 1 | 6/883 | 30/5950 | - | 1.35 [0.56, 3.25] |  | Muanda (2017)(2).25 |
|  | Clarithromycin | 1 | 4/658 | 30/5950 | - | 1.21 [0.42, 3.44] |  | Muanda (2017)(2).26 |
|  | Erythromycin | 1 | 4/697 | 30/5950 | - | 1.14 [0.40, 3.24] |  | Muanda (2017)(2).27 |
| Malformation: Cleft palate/lip | Azithromycin | 1 | 2/883 | 5/5950 | - | 2.70 [0.52, 13.93] |  | Muanda (2017)(2).31 |
|  | Clarithromycin | 1 | 2/658 | 5/5950 | - | 3.62 [0.70, 18.72] |  | Muanda (2017)(2).32 |
|  | Erythromycin | 1 | 3/697 | 5/5950 | - | 5.14 [1.23, 21.55] |  | Muanda (2017)(2).33 |
| Malformation: Craniosynostosis | Azithromycin | 1 | 8/883 | 27/5950 | - | 2.01 [0.91, 4.43] |  | Muanda (2017)(2).40 |
|  | Clarithromycin | 1 | 4/658 | 27/5950 | - | 1.34 [0.47, 3.85] |  | Muanda (2017)(2).41 |
|  | Erythromycin | 1 | 3/697 | 27/5950 | - | 0.95 [0.29, 3.13] |  | Muanda (2017)(2).42 |
| Malformation: Pyloric stenosis | Azithromycin | 1 | 7/1574 | 89/34222 | - | 1.71 [0.79, 3.69] |  | Lund (2014).3 |
|  | Clarithromycin | 1 | 1/223 | 89/34222 | - | 1.72 [0.24, 12.38] |  | Lund (2014).4 |
|  | Erythromycin | 2 | 10/6372 | 95/43332 | 72.8 | 1.01 [0.22, 4.70] |  | Lund (2014).5, Kallen (2005).3 |
