# Supplementary material for: Associations between use of macrolide antibiotics during pregnancy and adverse child outcomes: A systematic review and meta-analysis
Source: PLoS One. 2019 Feb 19;14(2):e0212212. doi: 10.1371/journal.pone.0212212 (PMC6380581; doi:10.1371/journal.pone.0212212)
Supplement: S4 Fig — (DOCX) [file pone.0212212.s012.docx]

**S4 Fig. Primary analysis (RCTs) for the association between adverse child outcomes and prenatal use of macrolides versus alternative antibiotics.**


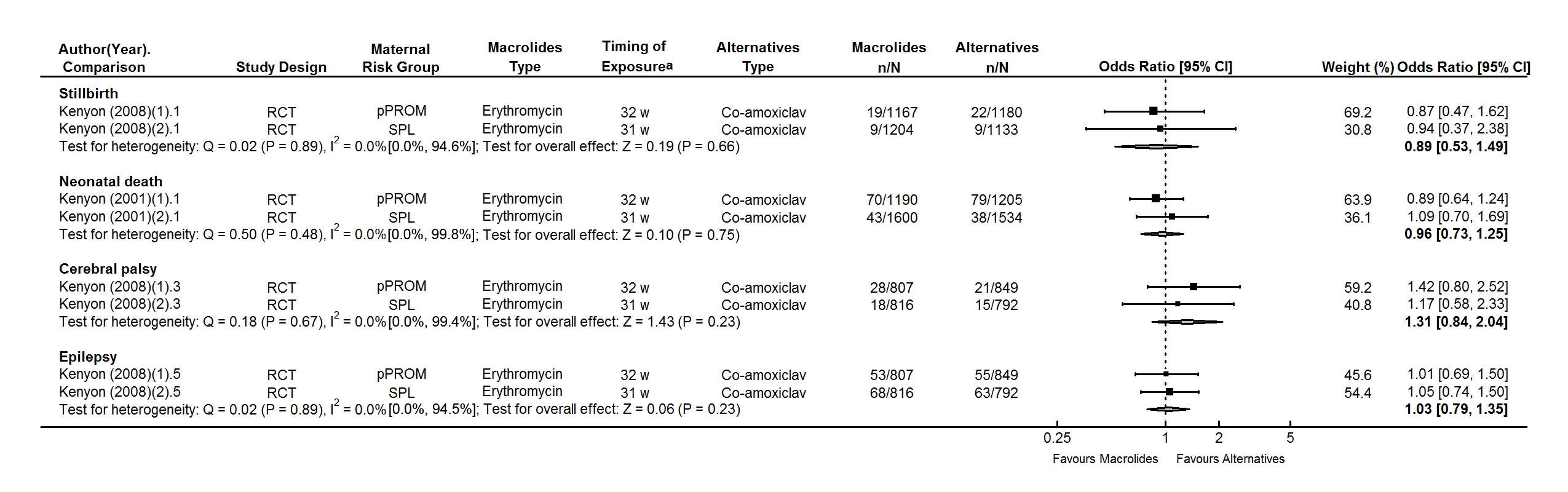


a. Priority of timing was given to median gestation age of exposure or randomisation, followed by mean, range and approximate time window of exposure; w: gestational week. pPROM: Preterm premature rupture of membranes; SPL: spontaneous preterm labour.
