# Supplementary material for: Associations between use of macrolide antibiotics during pregnancy and adverse child outcomes: A systematic review and meta-analysis
Source: PLoS One. 2019 Feb 19;14(2):e0212212. doi: 10.1371/journal.pone.0212212 (PMC6380581; doi:10.1371/journal.pone.0212212)
Supplement: S5 Fig — (DOCX) [file pone.0212212.s013.docx]

**S5 Fig. Secondary analysis (RCTs) for the association between adverse child outcomes and prenatal use of macrolides versus no macrolides.**


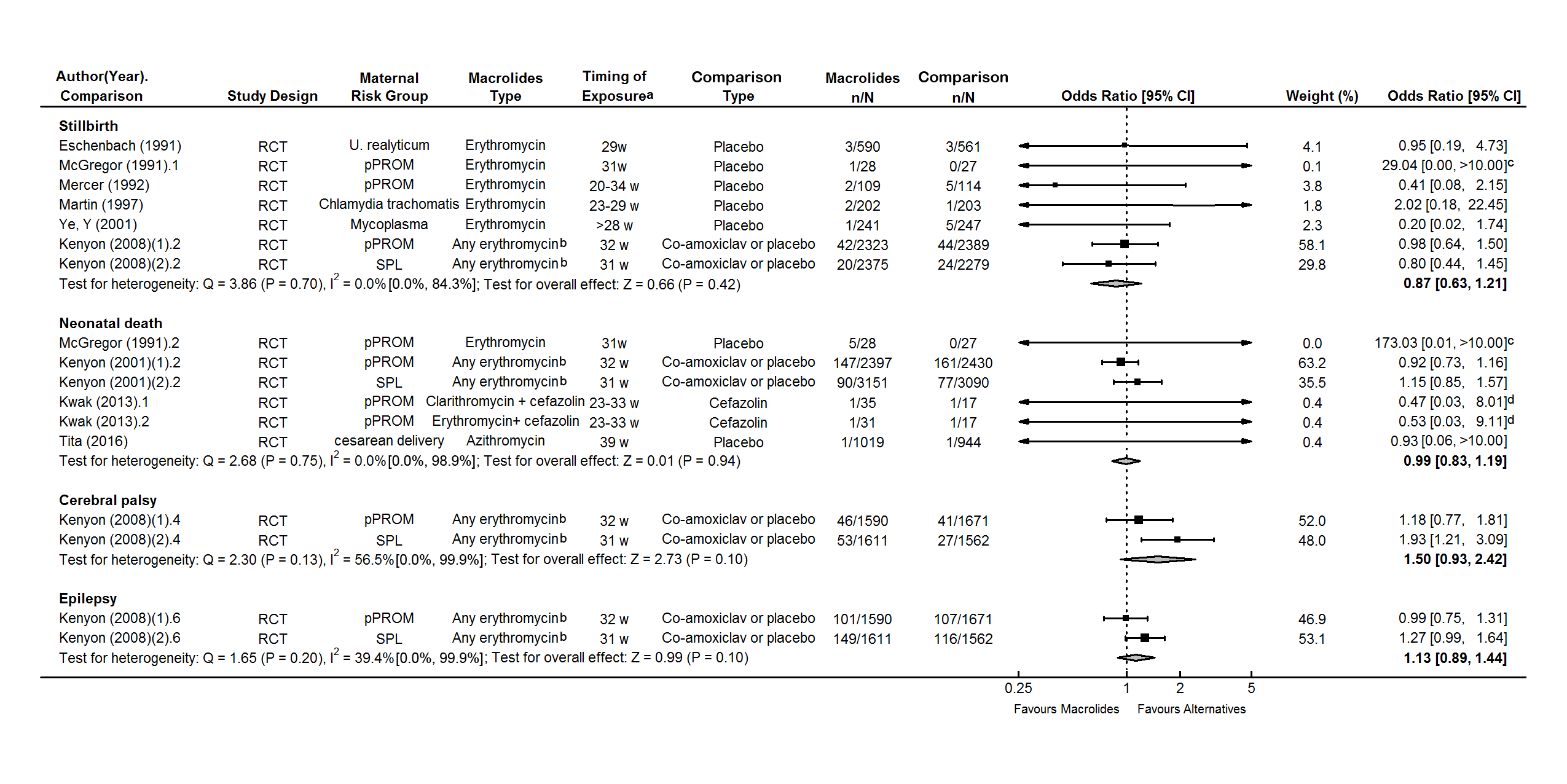


a. Priority of timing was given to median gestation age of exposure or randomisation, followed by mean, range and approximate time window of exposure; w: gestational week. b. Any erythromycin: erythromycin + co-amoxiclav or erythromycin only. c. In the study of McGregor (1991), the counts in erythromycin arm were adjusted by adding the reciprocal of the size of the opposite treatment arm size (1/27) and placebo arm adjusted by adding 1/28, due to zero event. d. In the study of Kwak (2013), participants in the cefazolin group (n = 34, including 2 events) were evenly split into 2 comparison groups to avoid double-counting.
