# Supplementary material for: Associations between use of macrolide antibiotics during pregnancy and adverse child outcomes: A systematic review and meta-analysis
Source: PLoS One. 2019 Feb 19;14(2):e0212212. doi: 10.1371/journal.pone.0212212 (PMC6380581; doi:10.1371/journal.pone.0212212)
Supplement: S6 Fig — (DOCX) [file pone.0212212.s014.docx]

**S6 Fig. Subgroup analysis: pooled results for specific macrolide types (RCTs, macrolides versus no macrolides).**

| Outcomes | Subgroup | Number of Studies | Macrolides n/N | No Macrolides n/N | I^2^ | Pooled OR  (95% CI) | Pooled Odds Ratio (95% CI) | Author (year).comparison number |
| --- | --- | --- | --- | --- | --- | --- | --- | --- |
| Stillbirth | Erythromycin | 7 | 71/5868 | 82/5820 | 0 | 0.87 [0.63, 1.21] |  | * |
| Neonatal death | Azithromycin | 1 | 1/1019 | 1/944 | 0 | 0.93 [0.06, 14.83] |  | Tita (2016) |
|  | Clarithromycin | 1 | 1/35 | 1/17 | 0 | 0.47 [0.03, 8.01] |  | Kwak (2013).1 |
|  | Erythromycin | 4 | 243/5607 | 239/5564 | 0 | 1.00 [0.83, 1.20] |  | McGregor (1991).2, Kwak (2013).2, Kenyon (2001)(2).2, Kenyon (2001)(1).2 |
| Cerebral palsy | Erythromycin | 2 | 99/3201 | 68/3233 | 56.49 | 1.50 [0.93, 2.42] |  | Kenyon (2008)(2).4, Kenyon (2008)(1).4 |
| Epilepsy | Erythromycin | 2 | 250/3201 | 223/3233 | 39.37 | 1.13 [0.89, 1.44] |  | Kenyon (2008)(2).6, Kenyon (2008)(1).6 |

*Ye, Y (2001), Mercer (1992), McGregor (1991).1, Martin (1997), Kenyon (2008)(2).2, Kenyon (2008)(1).2, Eschenbach (1991)
