# Supplementary material for: Associations between use of macrolide antibiotics during pregnancy and adverse child outcomes: A systematic review and meta-analysis
Source: PLoS One. 2019 Feb 19;14(2):e0212212. doi: 10.1371/journal.pone.0212212 (PMC6380581; doi:10.1371/journal.pone.0212212)
Supplement: S7 Fig — (DOCX) [file pone.0212212.s015.docx]

**S7 Fig. Sensitivity analysis according to risk of bias (based on primary analysis).**


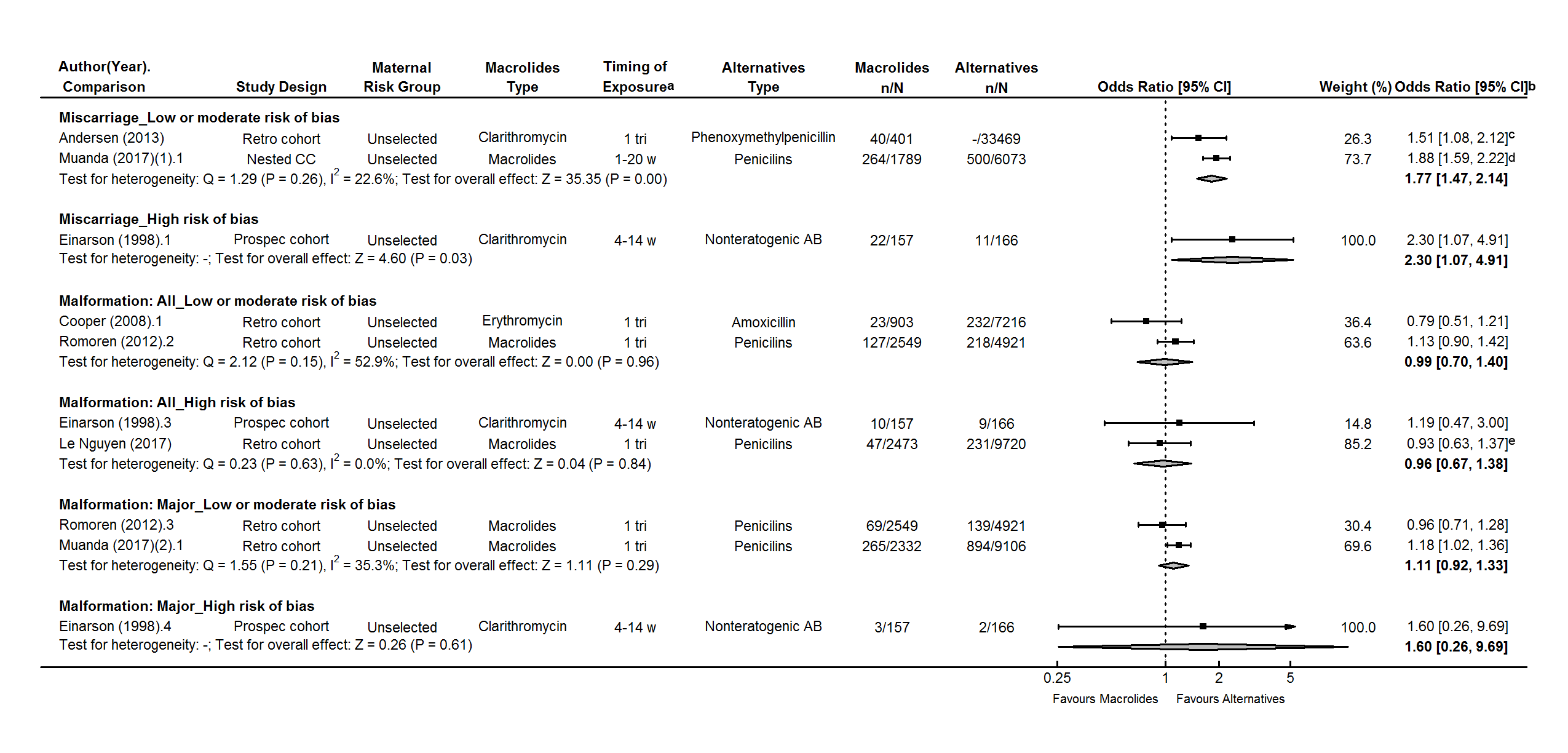


a. Priority of timing was given to median gestation age of exposure or randomisation, followed by mean, range and approximate time window of exposure; w: gestational week. b. Adjusted odds ratio/ hazard ratio was also shown if available. c. In the study of Andersen (2013), OR was adjusted by maternal age, number of previous miscarriages, income and education. Number of miscarriage in comparison group not given. d. In the study of Muanda (2017), cases and controls were matched by gestational age and year of pregnancy; OR were adjusted by 11 covariates, e.g. maternal age, education level, chronic comorbidities, maternal infections (urinary tract infection, respiratory tract infection, bacterial vaginosis and sexually transmitted infections) and prior exposure to antibiotics. e. In the study of Le guyen, OR was adjusted by maternal age, long-term illnesses, parity and multiple pregnancy. OR: Odds Ratio; CC: case control; AB: antibiotics.
